# Recognition of Cu^2+^ and Al^3+^ by a Quinolinyl 1,2,3-Triazole Chemosensor: A Comparative Study

**DOI:** 10.3390/s26144508

**Published:** 2026-07-15

**Authors:** Richard D. Govan, Tyler C. Camp, Vincent F. Hernandez, Precious Obiako, Debosreeta Bose, Debanjana Ghosh, Shainaz M. Landge, Karelle S. Aiken

**Affiliations:** 1 Department of Biochemistry, Chemistry, and Physics, Georgia Southern University, 521, College of Education Drive, Statesboro, GA 30460, USA; 2 Department of Basic Science and Humanities, Institute of Engineering and Management, University of Engineering and Management, New Town, Kolkata 700160, West Bengal, India; debosreeta.bose@iem.edu.in; 3 Department of Chemistry, Science Building West, Southern Illinois University Edwardsville, P.O. Box—1652, Edwardsville, IL 62026, USA

**Keywords:** 1,2,3-triazole, quinoline, copper (II), aluminum (III), fluorescence, colorimetric

## Abstract

1,2,3-Triazole units with their structural and photophysical properties are well-suited for the development of chemosensors for ion sensing. Synthetic approaches make it extremely easy to modify this core with just a few steps to control ion selectivity and response-signal output. The current study examines how 8-(4-phenyl-1*H*-1,2,3-triazol-1-yl)quinoline, a quinoline–triazole–phenyl (QTP) construct, responds differentially to Cu^2+^ and Al^3+^ ions. **QTP** provides distinct fluorescent signals in acetonitrile in the presence of Cu^2+^ versus Al^3+^, a turn-off response with Cu^2+^ and blue-to-green output with Al^3+^. Spectroscopic studies quantify the selectivity of the sensor for these species with respect to other ions and reveal a stoichiometric ratio of 1:1 for sensor:Cu^2+^ and 2:1 for sensor:Al^3+^. NMR titration studies suggest that Cu^2+^ is detected via coordination of the quinolinyl and triazolyl nitrogens, while Al^3+^ is detected through coordination of the quinoline nitrogen. Overall, **QTP** displays a selectivity for Al^3+^ relative to Cu^2+^ over other cations in this investigation.

## 1. Introduction

Cations are of fundamental importance to environmental and physiological processes. While an ion may be essential and advantageous in certain situations, the same ion can be considered xenobiotic or even an indicator of harmful conditions under different circumstances [1]. As such, ion imbalance, including the accumulation of excessive amounts, can have devastating effects.

Copper is important to ATP biosynthesis, proper brain function, and antioxidant processes [2]. Aluminum, the third most abundant metal on earth, is incorporated into materials for several important purposes: for example, water purification systems, food containers, machine parts, and construction [3,4,5]. Over-accumulation of either metal, copper, or aluminum, is harmful. For example, both are linked to the progression of neurodegenerative diseases such as Alzheimer’s and Parkinson’s disease [6]; amassing copper in the liver and brain leads to the debilitating effects of Wilson’s Disease, and the buildup of aluminum in the environment threatens aquatic and plant life [7,8]. For example, both are linked to the progression of neurodegenerative diseases such as Alzheimer’s and Parkinson’s disease [6,9]. Amassing copper in the liver and brain leads to the debilitating effects of Wilson’s Disease, and the buildup of aluminum in the environment threatens aquatic and plant life [10,11]. The development of inexpensive, ion-specific chemosensors to easily detect and quantify these species can facilitate a better understanding of their role in diseases and how they affect the environment [12].

The multifunctionality of the 1,2,3-triazole unit is excellent for sensing applications in colorimetric and fluorometric chemosensors [13]. Photophysical and structural properties of this heterocycle make it suitable for use as a part of the receptor, as a signaling unit, or as a linker connecting binding and reporter sites. As a receptor for cations, triazoles are N-donors through the π-bonded nitrogens [14,15,16]. The triazoles, by virtue of the synthetic procedures used to make them, are relatively easy to modify. The syntheses tend to be tolerant of several functional groups and high-yielding [17,18,19,20]. Facile modification of the substituents on triazoles can serve two important purposes: the ability to dictate (1) the cation selectivity and (2) tune the fluorometric or colorimetric signal output. In the current study, functionalizing the triazole unit with a quinoline moiety provides an extensively conjugated structure that can function as a fluorometric chemosensor for the Cu^2+^ and Al^3+^ ions.

**Scheme 1:**
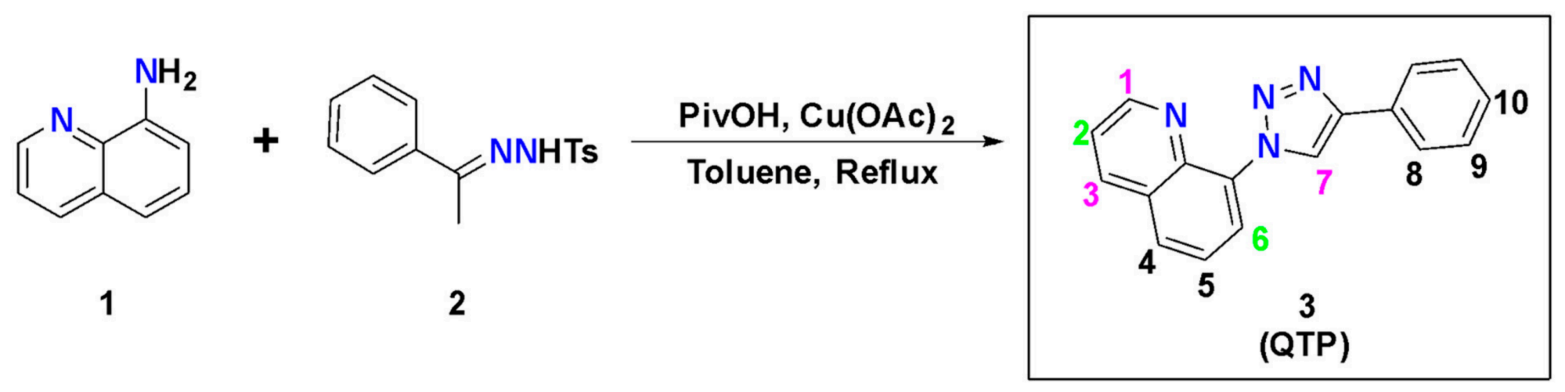
Synthesis of 8-(4-phenyl-1H-1,2,3-triazol-yl)quinoline, **QTP**.

Herein, we report on the contrast in the response of the 8-(4-phenyl-1*H*-1,2,3-triazol-1-yl)quinoline, a **q**uinoline-**t**riazole-**p**henyl (**QTP**) construct to Cu^2+^ versus Al^3+^, two of the ions that induce visible fluorometric changes in the molecule. **QTP** is known [17] but, to the best of our knowledge, this is the first report on its versatility as a chemosensor. The sensor is made by the coupling of 8-aminoquinoline and acetophenone *N*-tosylhydrazone in a copper (II)-mediated reaction (Scheme 1) [17]. With extensive conjugation throughout the structure and donor nitrogens in the triazolyl and quinolinyl units, **QTP** has cation-sensing capabilities that provide clear and distinctly different fluorescence-signal outputs for Cu^2+^ versus Al^3+^ under ultraviolet (UV) illumination at 356 nm—a complete “turn-off” response for Cu^2+^ and a “blue-to-green” output for Al^3+^. Nuclear Magnetic Resonance (NMR), UV-Vis absorption, and fluorescence spectroscopy were used to investigate the interaction of the probe with the cations and to decipher the difference in the binding mode between the two. Our results reveal a stoichiometric ratio of 1:1 for Cu^2+^ and 2:1 for Al^3+^ (**QTP**:metal).

## 2. Materials and Methods

### 2.1. General Experimental

All chemicals and reactants were obtained through commercial sources (Alfa Aeser, Ward Hill, MA 01835, USA; OXCHEM, Dallas, TX 75254, USA; Sigma-Aldrich, St. Louis, MO 63103, USA; Acros, Irvine, CA 92618, USA; and Fisher, Waltham, MA 02451, USA) without further purification. HPLC-grade solvents and de-ionized (DI) water were used for spectroscopic experiments and syntheses. Column chromatography was performed with Selecto Scientific Silica Gel (particle size 100–200 microns).

### 2.2. Synthesis of 8-(4-Phenyl-1H-1,2,3-triazol-yl)quinoline (QTP)

8-(4-Phenyl-1H-1,2,3-triazol-yl)quinoline (**QTP**) was synthesized according to a previously reported procedure (Scheme 1) [17]. Structural modifications in QTP resulting from the presence of metal ions were characterized via ^1^H-NMR (Nuclear Magnetic Resonance (NMR)) titration experiments. To ensure accurate assignment of the overlapping or shifted proton signals, two-dimensional ^1^H-^1^H COrrelation SpectroscopY (COSY) was subsequently employed (Figure S1A,B).

### 2.3. Determination of Stoichiometry (Job’s Plot) Between Sensor and Metal Ions

For Job’s plot, the product of the difference in fluorescence intensity (ΔF) and the mole fraction (Χ), ΔF^.^Χ, was plotted against Χ. ΔF is determined from F_0_ − F_x_, where F_0_ and F_x_ are the fluorescence intensities of **QTP** in the absence and presence of the metal ions (M^n+^). The maximum of the plot indicated the mole ratio between the interacting species.

### 2.4. Calculation of the Limit of Detection (LOD)

The detection limit of **QTP** for the metal ion, Cu^2+^, was calculated using the fluorescence titration according to the IUPAC definition (Equation (1)). The fluorescence of **QTP** was measured independently ten times and the standard deviation (σ) of the blank measurement was determined. To find the slope (*k*), the ratio of fluorescence intensities (F_x_/F_0_) at 415 nm was plotted against [Cu^2+^].

Limit of Detection (LOD) = 3σ/k(1)

### 2.5. Instrumentation

NMR spectra were recorded on an Agilent MR4000DD2 spectrometer (Agilent Technologies, Loveland, CO 80537, USA, originally manufactured by Varian Inc., Palo Atlo, CA 94304, USA) with a multinuclear probe with two RF channels and variable temperature capability, ^1^H-NMR: 400 MHz and ^13^C-NMR:100 MHz using deuterated acetonitrile (CD_3_CN). Signals were recorded in parts per million (*ppm*). References in the corresponding solvents were set according to residual CH_3_CN at 1.94 ppm for ^1^H-NMR and 1.32 ppm [CH_3_] and 118.26 [CN] for ^13^C-NMR. Signals for the ^1^H-NMR multiplicity are described as: singlet (*s*), doublet (*d*), doublet of doublet (*dd*), triplet (*t*), multiplet (*m*), coupling constants (*J*, Hz) and integration. Melting points were measured with the Vernier Melt Station using Vernier LabQuest 2 and are uncorrected.

Room temperature absorption and steady-state fluorescence measurements were performed using a Shimadzu UV-2450 spectrophotometer (Shimadzu Scientific Instruments, Columbia, MD 21046, USA, manufactured in Kyoto 604-8511, JPN) and a PerkinElmer LS55 (PerkinElmer, Inc., Shelton, CT 06484, USA, manufactured in Buckinghamshire, HP9 2FX, UK) with a well plate reader fluorimeter respectively. Concentrations of **QTP** were kept at 2.94 × 10^−4^ mol dm^−3^ or below in acetonitrile to avoid any possible intermolecular effect. The specific concentration for each experiment is identified in the corresponding figures.

## 3. Results and Discussion

The differential interactions of **QTP** with Cu^2+^ and Al^3+^ were investigated through UV illumination, UV–Vis absorption measurements, fluorescence analysis, and NMR spectroscopy. The behavior of the sensor with these cations was compared to a select group of metals to which the probe is essentially nonresponsive under UV-light (Ni^2+^, Cu^2+^, Cd^2+^, Zn^2+^, Li^+^, Al^3+^, Mg^2+^, Ag^+^). These investigations provided valuable insight into the interaction of the chemosensor with Cu^2+^ versus Al^3+^, such as the stoichiometry, the structural motifs involved in binding, and the ion selectivity of the molecule. Owing to the hydrophobic nature of QTP, the sensing mechanism was evaluated using non-coordinating perchlorate salts in organic media. While the probe in its current form is limited for direct aqueous anion-rich environments due to solubility constraints, future work will focus on structural modifications (e.g., appending hydrophilic groups) as also seen in our previous work [16].

### 3.1. Preliminary Observations Under UV-Light

**Figure 1:**
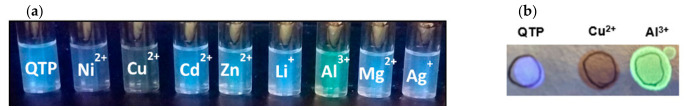
(**a**) Response of QTP (2.94 × 10^−4^ mol dm^−3^) treated with 10 equivalents of a diverse range of perchlorate salts in acetonitrile under UV-light (365 nm) and (**b**) photograph of the paper strip showcasing the real-life application of free sensor QTP, and QTP + Copper (II) and QTP + Aluminum (III) perchlorate salts at 365 nm.

At a molar ratio of 1:10, **QTP**: metal salt in acetonitrile, the response of the probe to the series of perchlorate salts of the cations was investigated under UV-light (365 nm) and ambient light (Figure 1a and Figure S2). Clear, visible changes, if any, only occurred under UV-light. The fluorescent blue of the probe was altered by Cu^2+^ and Al^3+^, a complete “turn-off” response with Cu^2+^ and a blue-to-green fluorescence signal with Al^3+^.

To investigate the practical sensing application of chemosensor **QTP**, paper test strips were prepared by spotting filter paper with **QTP** (free sensor) followed by the targeted cations (Cu^2+^ and Al^3+^). At 365 nm, the quenching of fluorescence with Cu^2+^ and the green output with Al^3+^ are clearly observed (Figure 1b).

### 3.2. Fluorescence Spectroscopic Response to Cu^2+^ and Al^3+^ Compared to Other Cations

**Figure 2:**
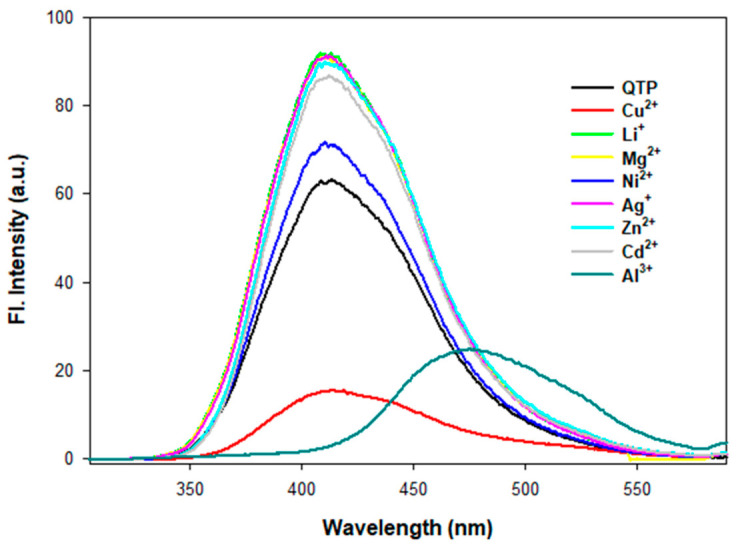
Fluorescence spectra of **QTP** sensor (2.94 × 10^−4^ mol dm^−3^) treated with perchlorate salts (~3 × 10^−3^ mol dm^−3^) in acetonitrile. *Exc*. at 294 nm.

The changes in the optical output of **QTP** with Cu^2+^ and Al^3+^ prompted an investigation of the photophysical behavior of the molecule with these ions. Fluorescence spectroscopy revealed that the maximum emission band for **QTP** occurs at ~411 nm with excitation at the maximum absorption of 294 nm (Figure 2 and Figure S3). Treatment with ten equivalents of the Cu^2+^ perchlorate reduced the intensity of this band by four-fold. The addition of Al^3+^ caused a bathochromic shift, from ~411 nm to ~477 nm, at an intensity that was 2.5-times lower than the original **QTP** maximum. The other salts enhanced the intensity at ~411 nm by different degrees but, visually, the corresponding response output for those salts was not as distinct as the changes induced by Cu^2+^ and Al^3+^ (Figure 1 and Figure 2).

Overall, screenings with the metal salts under UV-light and fluorescence spectroscopic studies revealed particularly distinct and contrasting responses with Cu^2+^ and Al^3+^. Studies were performed with these two cations to better understand the mechanism by which signaling occurs. All subsequent fluorescence experiments monitored bands at ~411/410 nm for Cu^2+^ and Al^3+^ in addition to ~477 nm for Al^3+^.

### 3.3. Cu^2+^: UV-Vis Absorption, Fluorescence, and NMR Spectroscopic Investigations

In-depth studies with fluorescence, absorbance and NMR experiments were used to understand the behavior of **QTP** with Cu^2+^.

#### 3.3.1. Cu^2+^: Investigation by UV-Vis Absorption

**Figure 3:**
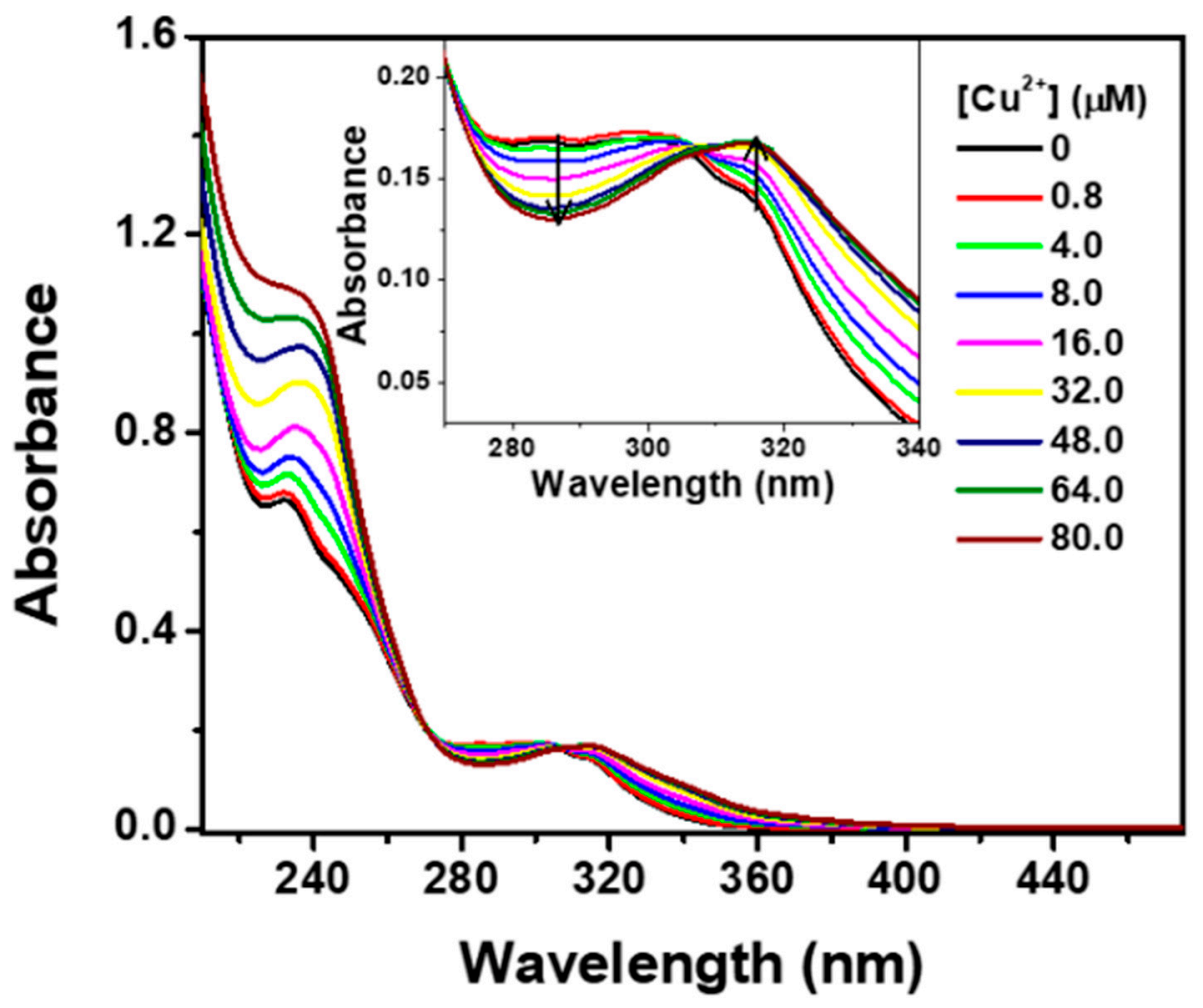
Absorbance spectrum of **QTP** (2 × 10^−5^ mol dm^−3^) with the addition (µL) of copper (II) perchlorate salt in acetonitrile. Vertical arrows indicate the direction in which changes occur with increasing amounts of the salt.

In acetonitrile, **QTP** exhibited an unstructured, low-energy absorption band peaking around 294 nm (Figure 3). Addition of the Cu^2+^ salt resulted in a gradual decrease in the 294 nm absorbance, followed by the emergence of a new band at 315 nm. This modulation in the absorption yielded two isosbestic points at 276 nm and 308 nm (Figure 3). The presence of the isosbestic points which occur due to an equilibrium between different species in a medium supports the formation of a **QTP**-Cu^2+^ complex. The appearance of the 315 nm band for this new species is due to the charge transfer from the triazolyl nitrogen to Cu^2+^ [21]. An intramolecular charge transfer (ICT) from the fluorophore to the Cu^2+^ occurs when **QTP** forms a stable complex with the metal ion. Other transitions, such as d-d, π-π*, and charge transfer (LMCT and MLCT) are also responsible for the change in the absorbance spectra of organic molecules with metal ions [22].

#### 3.3.2. Cu^2+^**:** Fluorescence Spectroscopic Investigation

**Figure 4:**
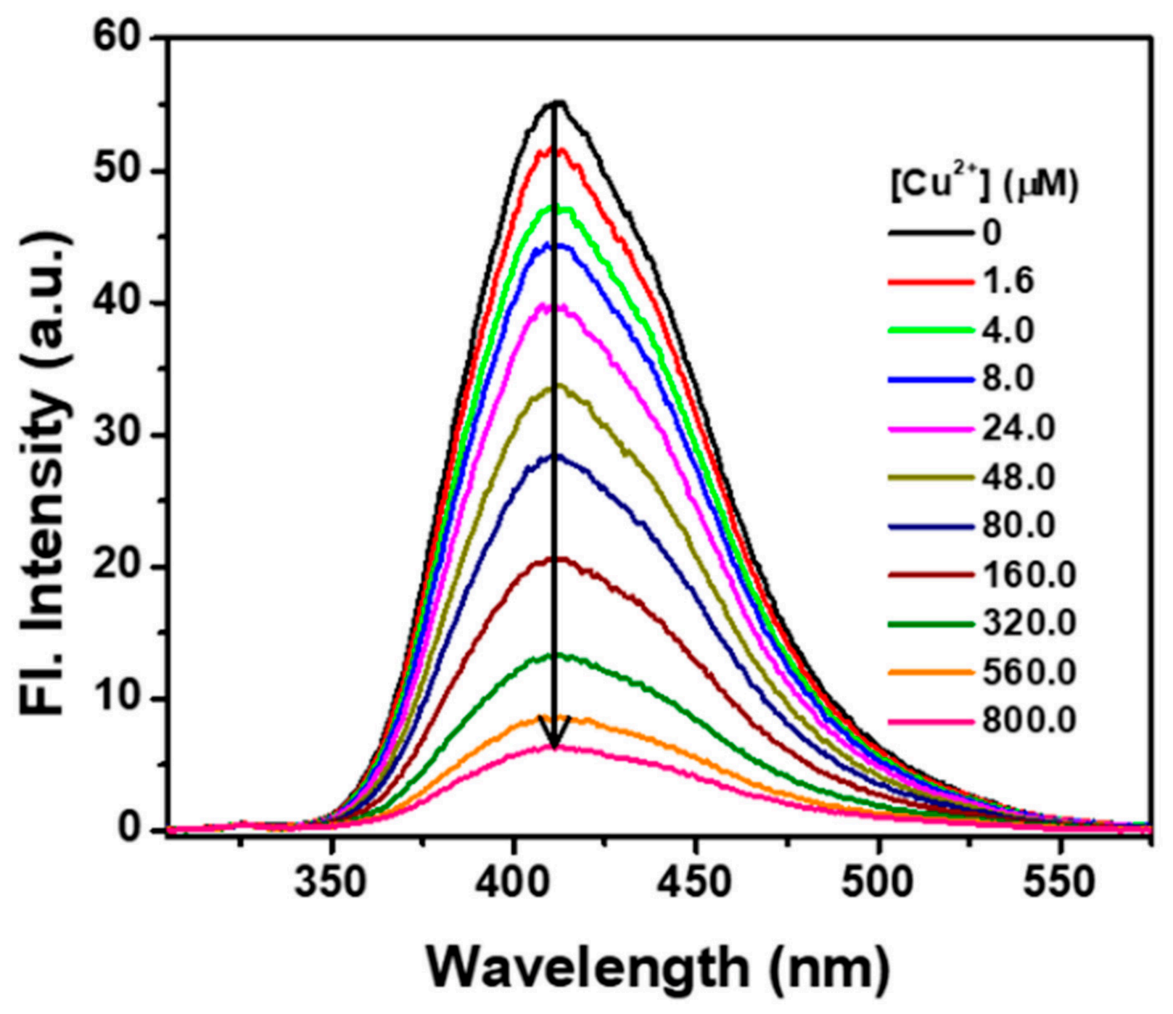
Fluorescence titration of the **QTP** sensor (2.95 × 10^−4^ mol dm^−3^) with the addition of copper (II) perchlorate hexahydrate in acetonitrile. *Exc*. at 294 nm. The vertical arrow indicates the direction in which changes occur with increasing amounts of the salt.

**Figure 5:**
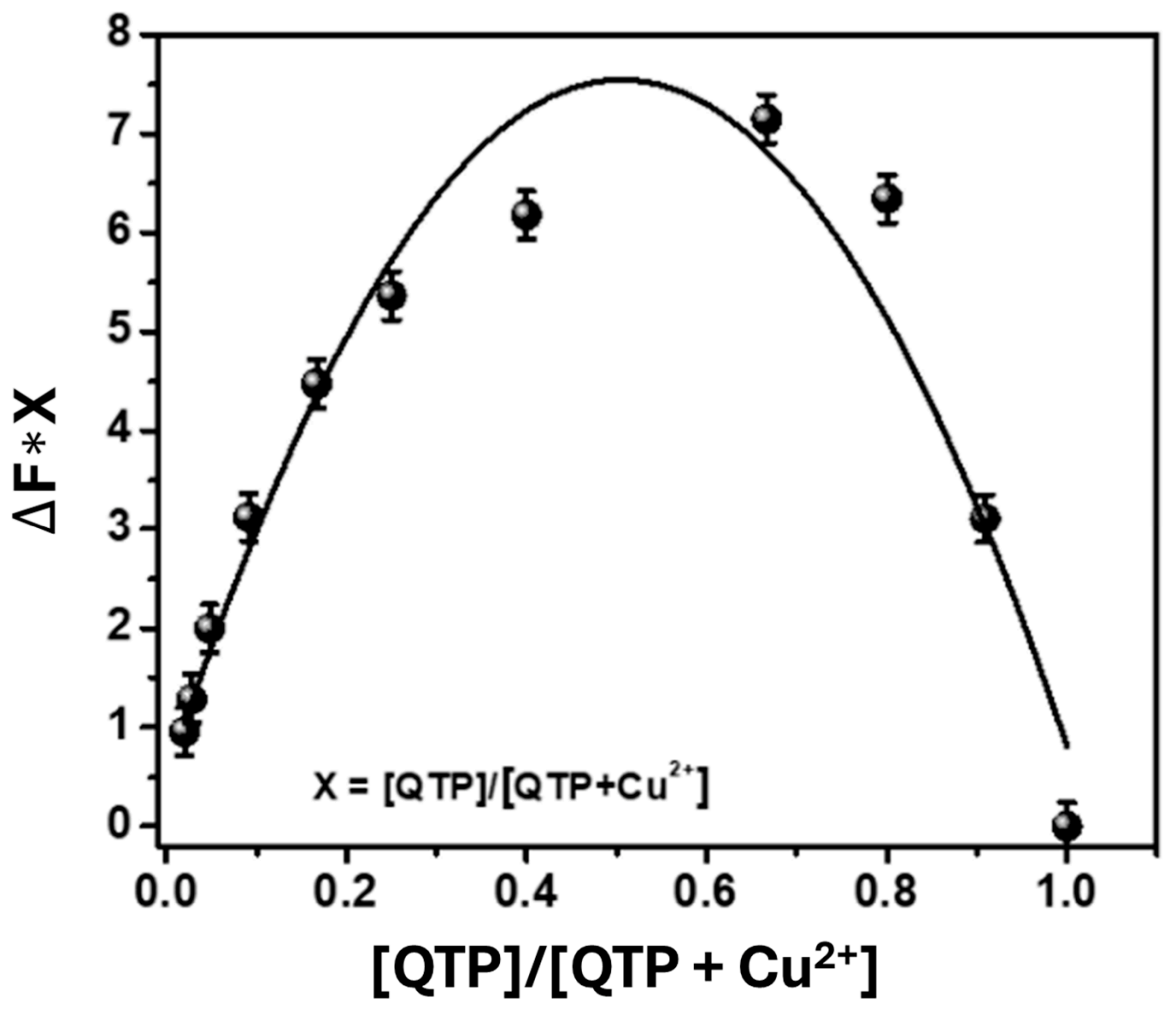
Job’s plot of **QTP** with copper (II) perchlorate salt in acetonitrile, emission monitored at 411 nm. *Exc*. at 294 nm.

A fluorescence titration study also revealed a 1:1 binding stoichiometry between Cu^2+^ and **QTP** (Figure 4 and Figure 5). Changes in the fluorescence at 411 nm were monitored while varying the equivalents of the perchlorate relative to the sensor. The chelation-enhanced fluorescence quenching (CHEQ) impact of paramagnetic Cu^2+^ ions produced a notable decrease in the fluorescence intensity of **QTP**. For the Job’s plot, the emission intensity was monitored at 411 nm. A non-linear curve fit parameter went through a maximum ΔF^.^Χ at Χ = 0.51 correlating with a 1:1 stoichiometric ratio for **QTP**:Cu^2+^ (Figure 5). A Job’s plot based on the change in absorbance at 315 nm (Figure S4) was consistent with the 1:1 stoichiometry. The binding constant according to the Benesi–Hildebrand plot for this interaction is 1.45 × 10^5^ M^−1^ (Figure S10) [23].

**Figure 6:**
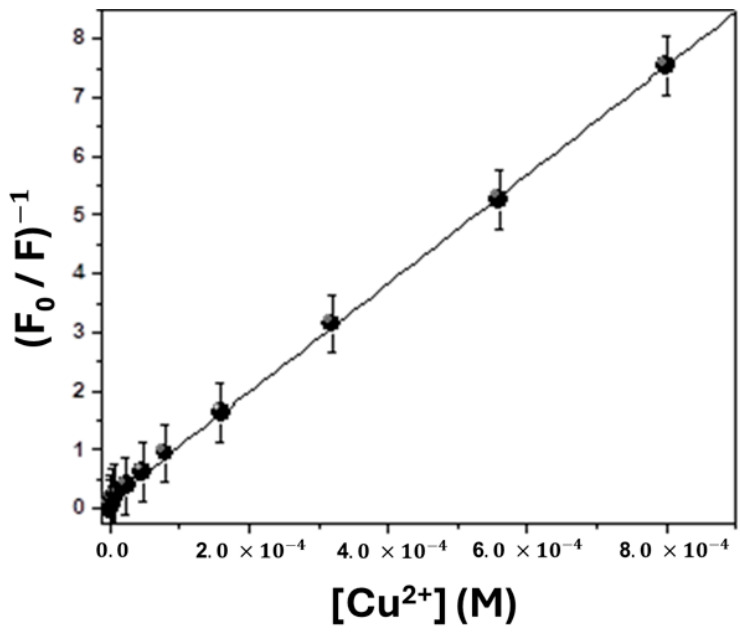
The Stern–Volmer plot for the fluorescence quenching of **QTP** in the presence of copper (II) perchlorate hexahydrate.

In order to understand the type of fluorescence quenching, collisional or dynamic, the decrease in the fluorescence intensity was plotted using the Stern–Volmer equation, F_0_/F = 1 + K_SV_ [Cu^2+^], where F_0_ and F are, respectively, the fluorescence intensities of **QTP** in the absence and presence of the Cu^2+^ (quencher) at 411 nm (Figure 6) [24]. A linear plot indicated dynamic quenching of **QTP** in the presence of Cu^2+^. K_SV_, the Stern–Volmer quenching constant, as revealed from the slope of the plot is 9.26 × 10^4^ M^−1^ which is higher than the order for diffusion-controlled collisional quenching (10^1^–10^2^ M^−1^) [25,26,27]. This strongly indicated that the quenching of **QTP** is caused by the complexation with Cu^2+^. The limit of detection (LOD) as revealed from the F_x_/F_0_ showed a linearity from 1.6 to 24 μM of copper (II) addition. At 1.6 μM, it is therefore possible to detect extremely low levels of Cu^2+^ with **QTP** using fluorescence spectroscopy. Furthermore, this LOD is in the range of previously reported values, between ~77nM and 2.5 µM, for other small-molecule quinoline-based Cu^2+^-sensors which bind 1:1, sensor:cation [28,29]. On par with other reported LODs, **QTP** has the added benefit of its one-step synthesis and distinct “turn-off” response with Cu^2+^.

#### 3.3.3. ^1^H-NMR Investigations with the QTP-Cu^2+^ Complex

**Scheme 2:**
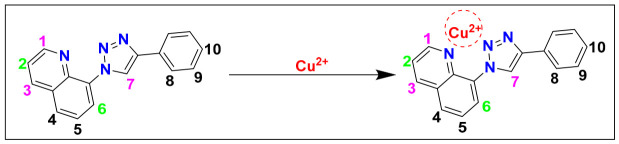
A schematic representation of the plausible interaction of **QTP** with Cu (II).

**Figure 7:**
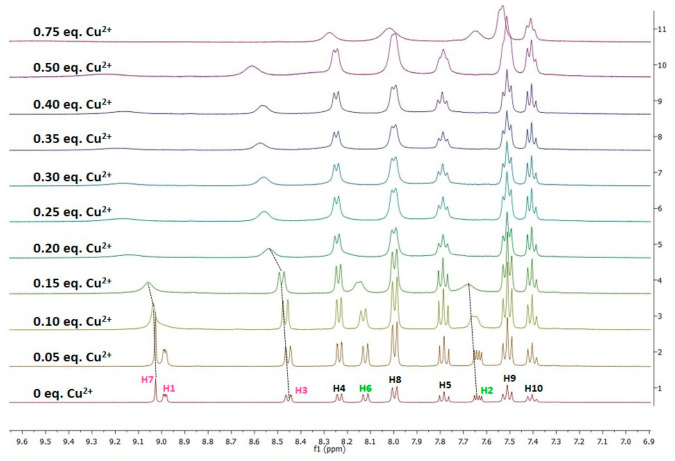
Titration experiments with **QTP** sensor (3.68 × 10^−2^ mol dm^−3^) and Cu (II) perchlorate salt in acetonitrile using ^1^H-NMR spectroscopy.

Details regarding the interactions in the Cu-**QTP** species were obtained using an ^1^H-NMR titration experiment with Cu^2+^ where the amount of **QTP** was held constant. These results clearly showed the participation of the triazolyl and quinolinyl nitrogens in the detection process [30]. Increasing the amount of Cu^2+^ resulted in downfield shifts for the triazole’s H-7 and the quinoline’s H-1 and H-3 (Figure 7). The maximum observable change occurred with H-3, from 8.45 ppm at 0.0 eq of Cu (II) to 8.60 ppm with 0.50 eq of Cu (II). The chemical shifts for H-1 and H-7 seemed to merge at ~9.10 ppm with 0.15 eq Cu^2+^, each originating at 8.99 and 9.04 ppm, respectively. The observed deshielding of protons whose environments depend on the electronic character of the ring nitrogens indicates that both the triazole (H-7) and quinoline (H-1 and H-3) nitrogens participate directly in coordinating to Cu^2+^. The triazolyl–quinolinyl binding-pocket model is strongly reinforced by the pronounced line broadening and eventual disappearance of proton signals. Protons are located in close proximity to the Cu^2+^ center, specifically H-2 and H-6 at 0.15 equiv. of Cu(II), followed by H-1 and H-7 at approximately 0.30 eq consistent with direct paramagnetic perturbation upon coordination [31]. Not much could be discerned at ≥0.75 eq due to broadening of the signals caused by the paramagnetism of Cu^2+^. However, the information obtained at lower equivalents clearly demonstrates the involvement of N-donor groups from both the quinoline and triazole in the formation of a probe-metal complex (Scheme 2).

### 3.4. Al^3+^: UV-Vis Absorption, Fluorescence, and NMR Spectroscopic Investigations

Spectroscopic investigations with fluorescence, UV-Vis absorption and NMR revealed the mechanism of response to Al^3+^.

#### 3.4.1. Al^3+^: Investigation by UV-Vis Absorption

The spectrophotometric titrations of **QTP** were performed with different concentrations of Al^3+^ salt in acetonitrile (Figure S5). Upon addition of Al^3+^, the absorption bands centered around 294 nm showed a gradual decrease in absorbance with the simultaneous development of a new peak around 345 nm. In addition to these changes, the high-energy band (~294 nm) showed red-shifted absorption. The preliminary observation of **QTP** with Al^3+^ in UV-Vis absorption prompted a quantitative analysis using fluorescence spectroscopy for the formation of the **QTP**-Al^3+^ complex.

#### 3.4.2. Al^3+^: Fluorescence Spectroscopic Investigation

**Figure 8:**
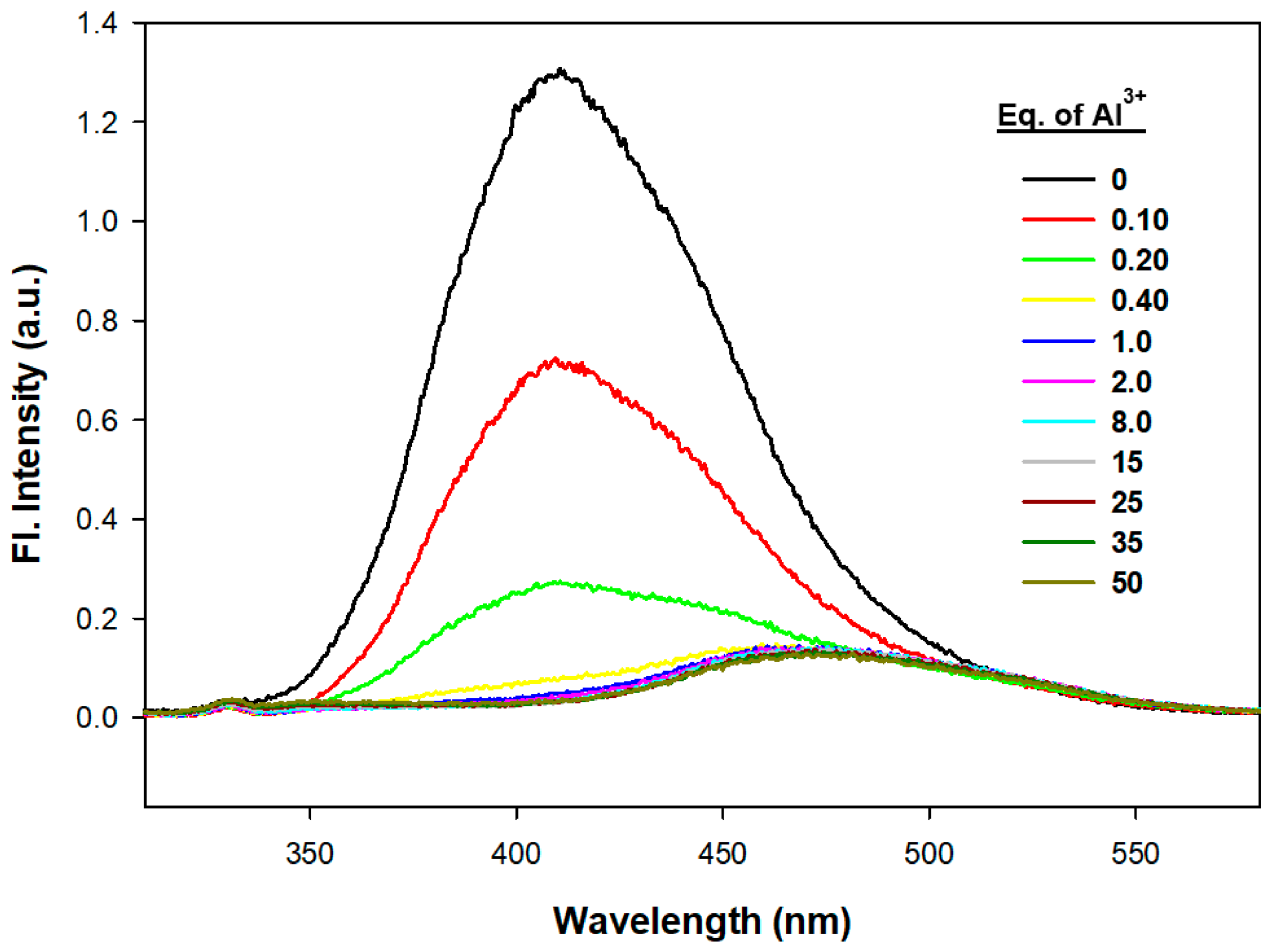
Fluorescence titration of the **QTP** sensor (1.47 × 10^−6^ mol dm^−3^) with the addition of Al^3+^ perchlorate hexahydrate (2.00 × 10^−5^ mol dm^−3^) in acetonitrile. *Exc*. At 294 nm.

As mentioned in the fluorescence screening study (Section 3.2), the emission of **QTP** yielded a peak around 411 nm. Fluorescence spectroscopy titration experiments with the **QTP** sensor and Al^3+^ showed a decrease in the emission band at ~411 nm until 0.3 eq of the metal with respect to **QTP** (Figure 8). Development of a new band around 477 nm was observed when 0.4 equivalents of Al^3+^ were added to **QTP**. This is consistent with the signal output observed under UV-light in Figure 1 which shows that Al^3+^ induces a blue to green color change with **QTP**. The ratiometric change in the emission of **QTP** is considered an outcome of the combined effect of the Lewis acidity of Al^3+^, solvation and the formation of the metal-ligand complex [32]. Quinoline probes have often shown modulation in the emission due to changes in local pH [33]. Asthana et al. [34] synthesized a potent fluorescent sensor, CMO for the detection of Al^3+^. Further, the CMO-Al^3+^ ensemble demonstrated effective performance as a selective probe for the detection of pyrophosphate (PPi) and picric acid. The results from their study revealed that the operational pH range of CMO for the detection of Al ^3+^ is 4–8.

**Figure 9:**
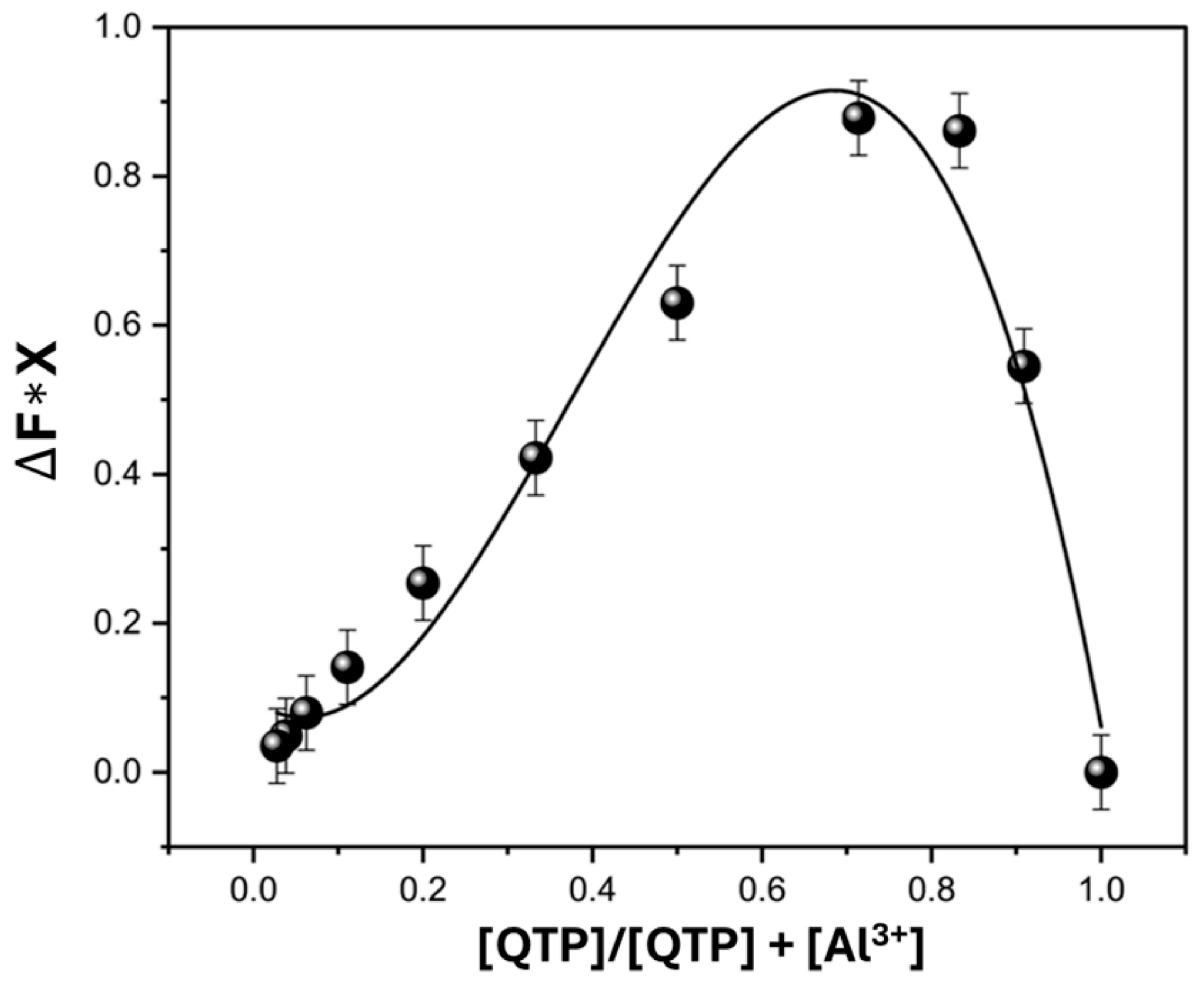
Job’s plot of **QTP** with Al^3+^ perchlorate salt in acetonitrile, emission monitored at 411 nm. *Exc*. at 294 nm.

Job’s plot based on the emission band intensity at 411 nm revealed a 2:1 stoichiometric ratio for the sensor: Al^3+^ ion (Figure 9). In this plot, the non-linear curve fit parameter went through a maximum ΔF^.^Χ at Χ = 0.70, correlating a 2:1 binding stoichiometry between **QTP** and Al^3+^. The binding constant based on the Benesi–Hildebrand plot is 1.42 × 10^6^ M^−2^ (Figure S11) [23]. The LOD based on the Stern–Volmer plot is 4.27 µM (Figure S6) somewhat higher than similar small-molecule sensors which bind 2:1, sensor:Al^3+^ with LODs in the ~70–80 nM range [35,36]. However, as noted previously, **QTP**’s one-step synthesis and its distinct response to the cation, in this case blue to green, are an advantage; this speaks to its potential for further development for practical use.

#### 3.4.3. ^1^H-NMR Investigations with the QTP-Al^3+^ Complex

**Scheme 3:**
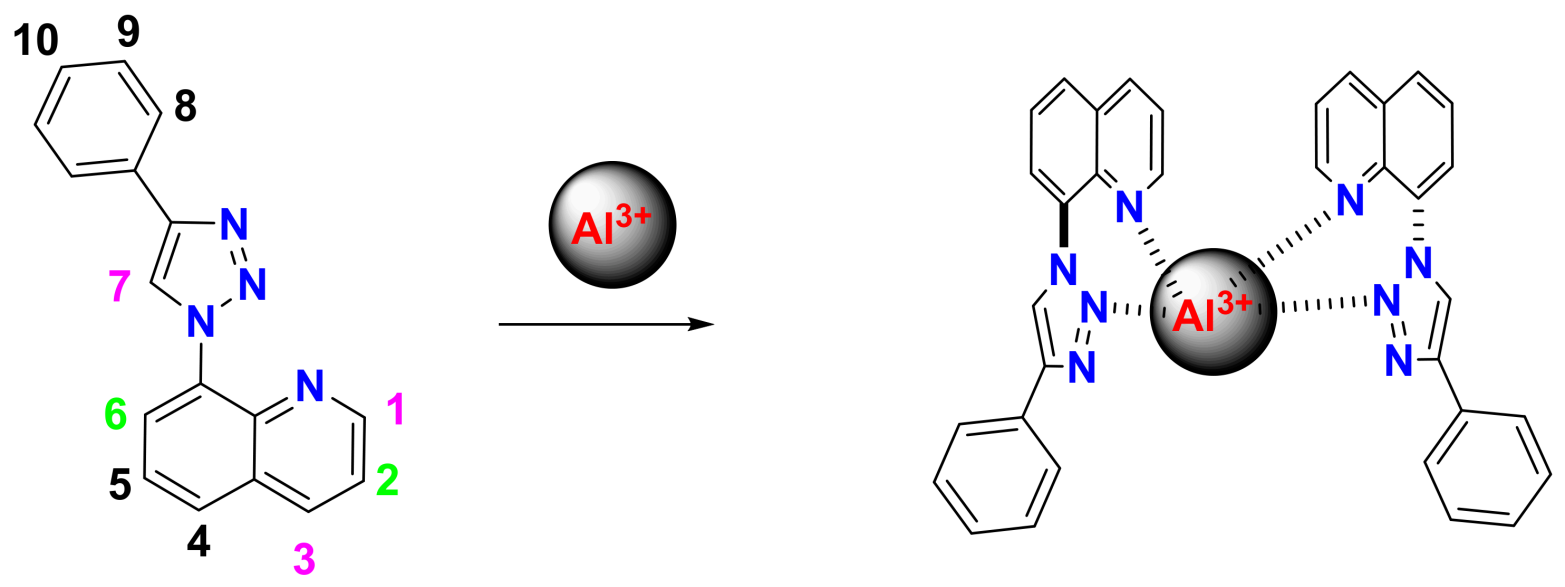
A schematic representation of the plausible interaction of **QTP** with Al (III).

**Figure 10:**
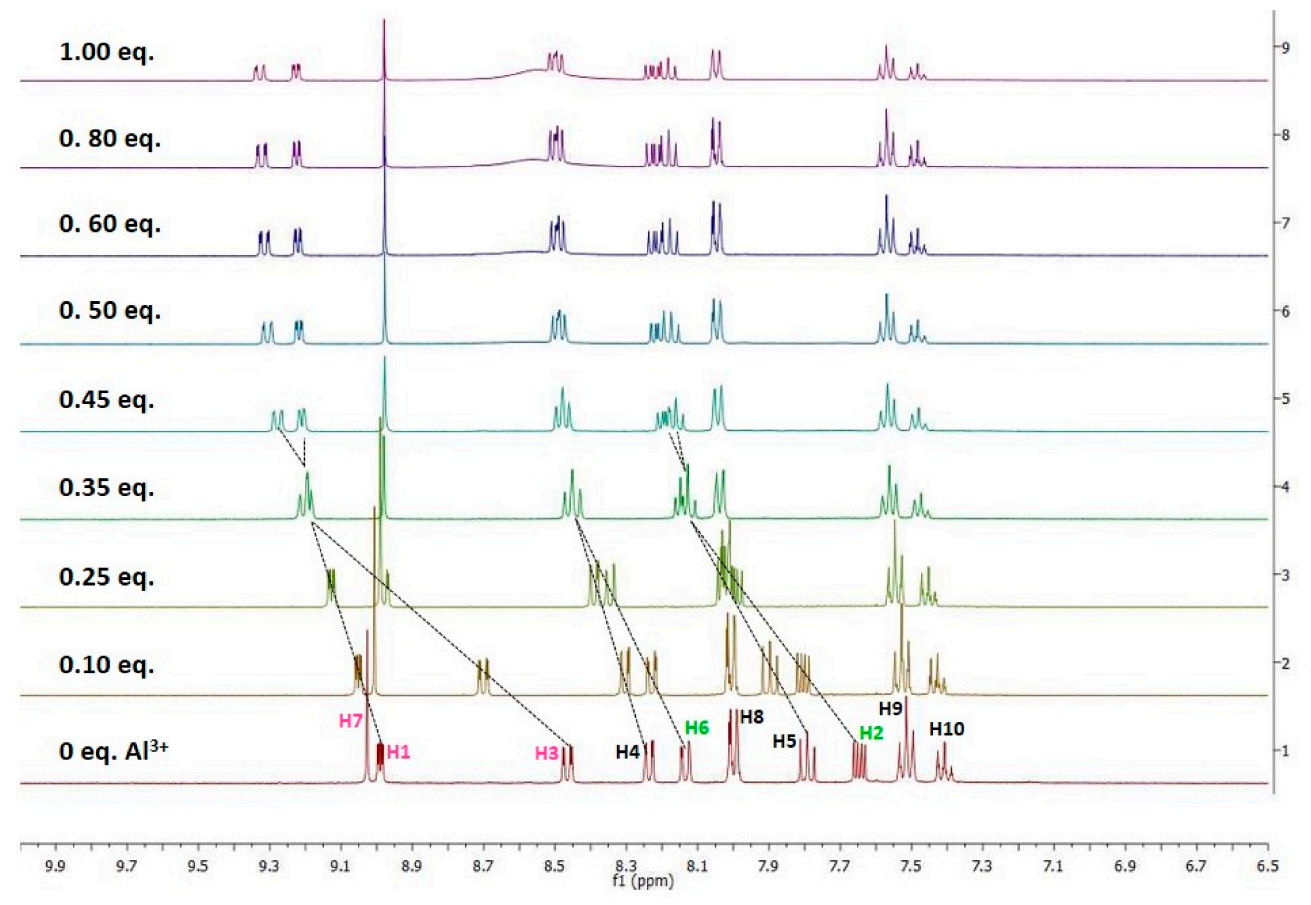
Titration experiments with **QTP** sensor (1.47 × 10^−3^ mol dm^−3^) and Al^3+^ perchlorate salt (1.00 × 10^−3^ mol dm^−3^) in acetonitrile using ^1^H-NMR spectroscopy.

NMR titration experiment with **QTP** and aluminum perchlorate strongly suggests that the sensor binds to Al^3+^ via its quinolinyl nitrogen (Figure 10) [37,38,39]. The behavior of the sensor with this ion is vastly different from that of Cu^2+^ (Figure 7). The change in the triazole proton’s resonance, H-7, is marginal compared to quinolinyl signals that are substantially shifted downfield between 0 and ~0.50 eq of Al^3+^ (Figure 10). With increasing amounts of Al^3+^, the resonances for H-1 and H-3 move from 8.99 ppm to 9.22 ppm, and 8.46 ppm to 9.32 ppm, respectively. The chemical shift for H-5 changes from 7.79 ppm to 8.18 ppm while signals for H-2 move from 7.65 ppm to 8.23 ppm. The peaks for H-4 at 8.24 ppm and H-6 at 8.14 ppm eventually merge and become centered around 8.50 ppm. In contrast, the triazole’s C_sp2_-H resonance, H-7, moves upfield by only 0.06 ppm from 9.04 ppm to 8.98 ppm. This slight upfield shift as opposed to a significant downfield movement in the H-7 signal strongly suggests that the triazolyl nitrogens are not involved in the binding to the Al^3+^ [38,39]. The phenyl signals, H-8, H-9 and H-10, remain unchanged regardless of the amount of Al^3+^ and after 0.6 eq, the quinolinyl signals stop responding to the addition of the cation. A Job’s plot based on the NMR titration corroborated the results of the fluorescence Job’s plot, with a maximum of ~0.7 that indicates a ratio of 2:1, **QTP**:Al^3+^ (Scheme 3, Figure 9 and Figure S7).

DFT calculations on the **QTP**–Al^3+^ coordinated geometries were performed using Gaussian 09W software with the ωB97XD functional and 6-311G(d,p) basis set using acetonitrile as solvent (Figures S8 and S9) [40]. The solvation was computed using the SMD (Solvation Model based on Density) model [41]. The optimized geometry suggests the participation of triazole nitrogen along with the quinolinyl nitrogen in binding Al^3+^ with **QTP** molecules as evidenced by their close proximities (Figures S8B and S9). In the optimized geometry, the close proximity of nitrogen atoms to Al^3+^, especially N_21_—Al_67_ and N_15_—Al_67_ points towards a nitrogen-assisted binding rather than an isolated one (Figure S9). NMR titration with Al^3+^ on **QTP** though shows little change in the triazole signal but this is not explicit of the excluded triazole nitrogen atom in binding with Al^3+^. Triazole nitrogen coordinated to Al^3+^ can induce localized electronic perturbations which might have little effect on the NMR signals as evidenced by the DFT calculations suggesting the triazole nitrogen and quinolinyl nitrogen bound **QTP**–Al^3+^ structure [42].

### 3.5. Interference Studies Probing the QTP’s Response to Cu^2+^ and Al^3+^ via Fluorescence

Interference studies to determine the sensitivity of the sensor compared to the other cations in this investigation were performed with Cu^2+^ and Al^3+^ cations.

#### 3.5.1. Interference Studies with Cu^2+^

**Figure 11:**
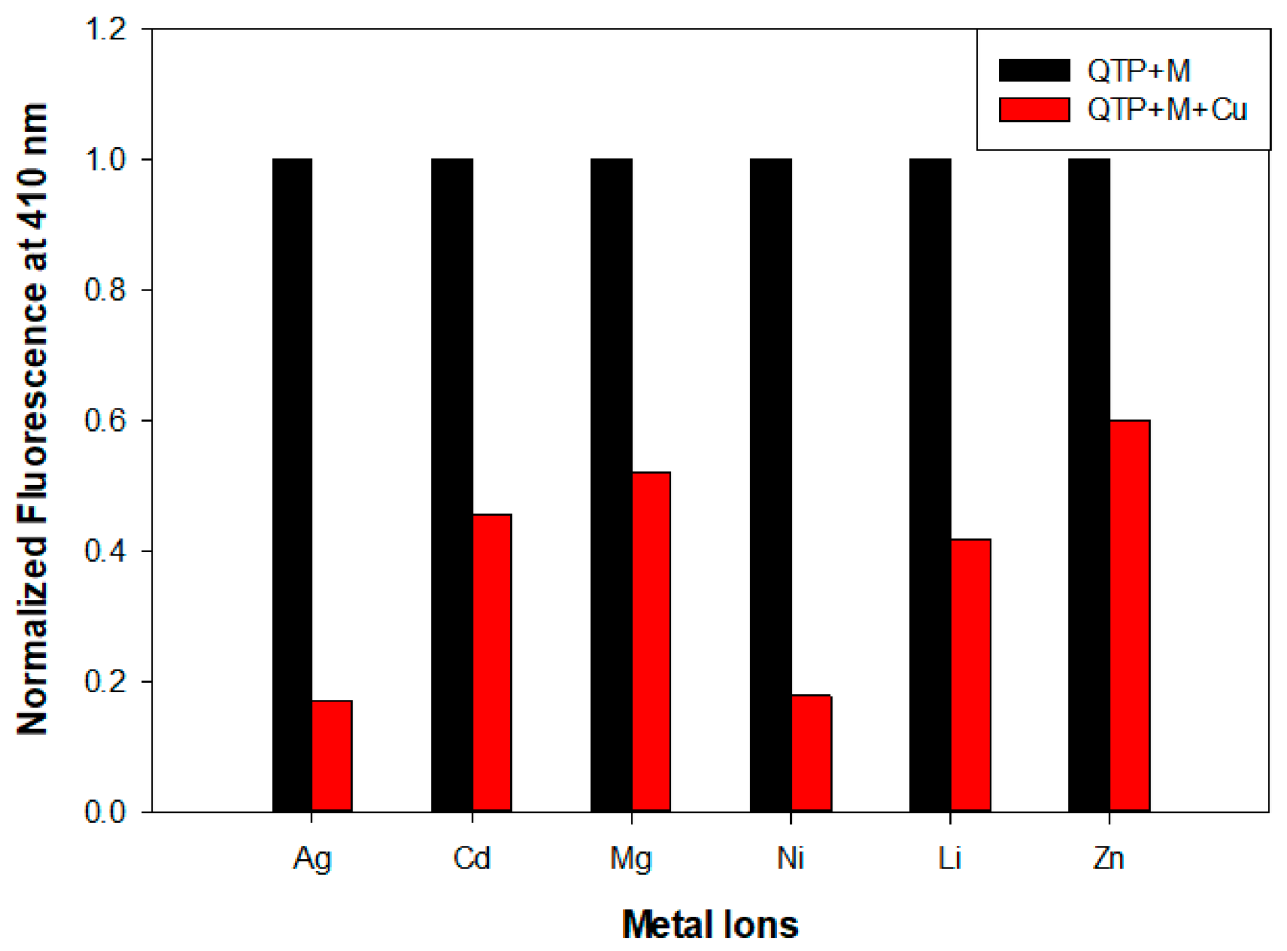
Interference experiments with **QTP** sensor with perchlorate salts of Ni^2+^, Cd^2+^, Zn^2+^, Mg^2+^, Li^+^, and Ag^+^ (10 eq for each) and Cu^2+^ (5 eq) with respect to **QTP**.

To varying degrees, five equivalents of Cu^2+^ muted the fluorescence of the sensor in the presence of ten equivalents of the Ni^2+^, Cd^2+^, Zn^2+^, Mg^2+^, Li^+^, and Ag^+^ ions (Figure 11). Significantly, in all cases, fluorescence intensity was reduced to 60% of the original value or less with the addition of copper (Figure 11). The most pronounced effect was observed with Ni^2+^ and Ag^+^ with which the addition of copper (II) reduced the fluorescence by more than 80%.

#### 3.5.2. Interference Studies with Al^3+^

**Figure 12:**
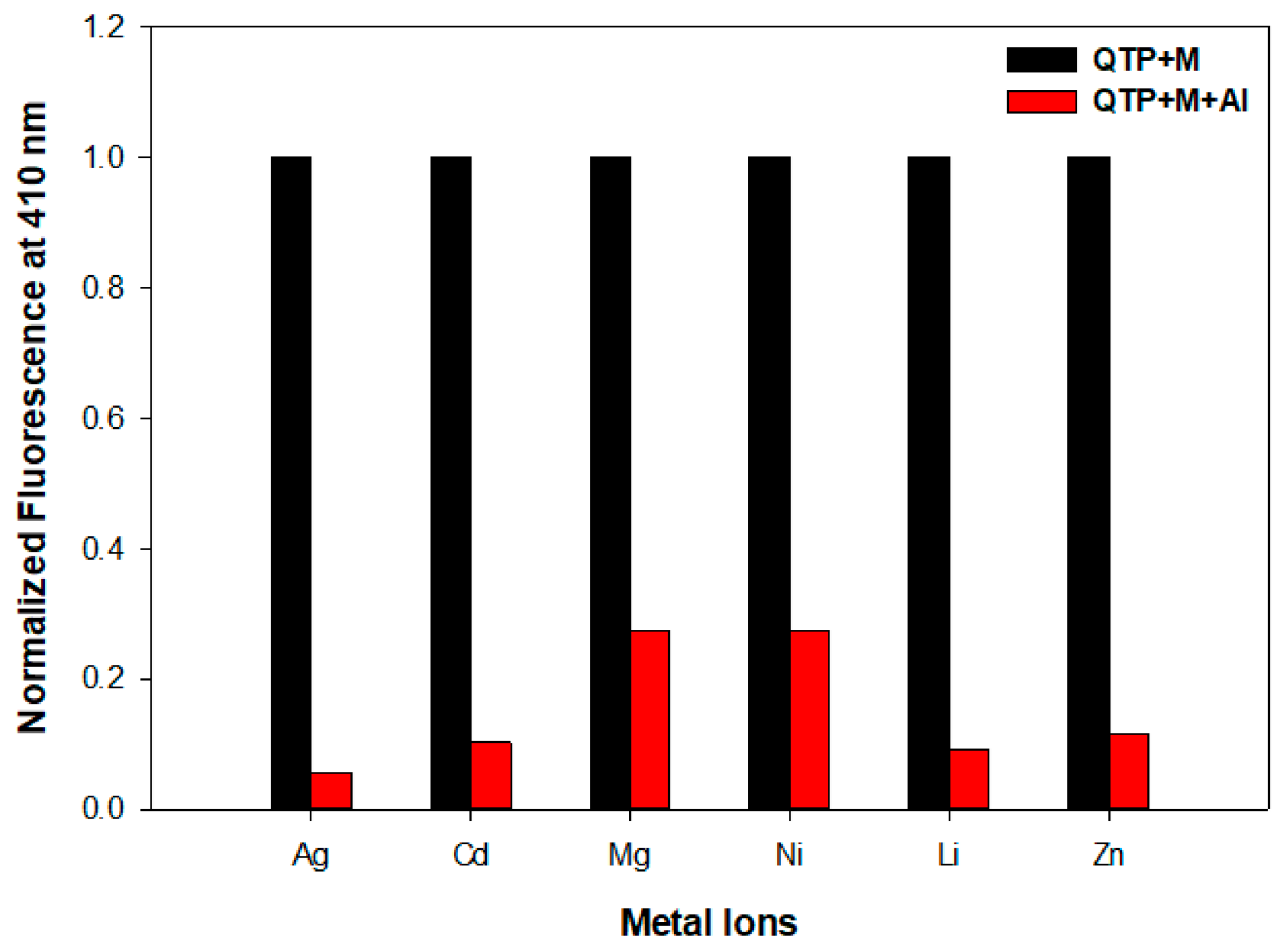
Interference experiments with **QTP** sensor with perchlorate salts of Ni^2+^, Cd^2+^, Zn^2+^, Mg^2+^, Li^+^, and Ag^+^ (10 eq for each) and Al^3+^ (5 eq).

For the aluminum perchlorate addition of 5 eq. of Al^3+^ to an acetonitrile solution of the sensor and 10 eq. of the interfering cations, Ni^2+^, Cd^2+^, Zn^2+^, Mg^2+^, Li^+^, and Ag^+^, showed a clear reduction in the intensity at ~410 nm (Figure 12). A comparison of the fluorescence output at 410 nm in the presence and absence of Al^3+^ indicates a distinct and strong preference for the Al^3+^ ion over the other metals, more so than was observed with Cu^2+^. In all cases, the 410 nm band was reduced to ~30% or less of its original value when Al^3+^ was added.

#### 3.5.3. Cu^2+^ Versus Al^3+^ Interference

**Figure 13:**
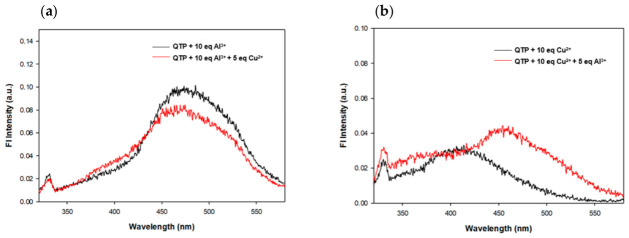
Interference experiment for **QTP** in acetonitrile with (**a**) 5 eq of copper (II) perchlorate added to 10 equivalents of aluminum perchlorate and the sensor and (**b**) 5 eq of aluminum (III) perchlorate added to 10 equivalents of copper (II) perchlorate and the sensor.

In exploring the preference of the sensor for Al^3+^ compared to Cu^2+^_,_ it was noted that a selectivity for Al^3+^ was somewhat maintained in the presence of Cu^2+^. The addition of 5 eq of the copper (II) perchlorate salt to a solution of the sensor and 10 eq of Al^3+^ reduced the intensity at 477 nm to ~80% of the initial value but did not revert to ~411 nm maximum that is observed when the sensor is only in the presence of Cu^2+^ (Figure 2 and Figure 13a). Doing the study in reverse, treating a mixture of the sensor and 10 eq. copper (II) with half the amount of Al^3+^, 5 eq., caused a red shift in the λ*_max._* from ~411 nm to ~460 nm. This movement toward the 477 nm band clearly shows a response that is specific to Al^3+^ and is most indicative of the aluminum cation’s ability to compete with Cu^2+^ for binding to **QTP**.

## 4. Conclusions

Under UV illumination (365 nm), **QTP**, a triazolyl-quinoline-based sensor, was found to produce a “turn off” response to Cu^2+^ and blue-to-green signal output for Al^3+^. NMR and DFT studies provided valuable information about the binding interaction. **QTP** appears to interact with Cu^2+^ via nitrogens in both the triazole and quinoline units based on NMR data while Al^3+^ strongly interacts with the molecule through the quinolinyl nitrogen as seen in NMR, and through the triazole N as predicted by DFT calculations. Job’s plots from fluorescence titration studies confirm a 1:1 stoichiometric ratio for **QTP**:Cu^2+^ and a 2:1 binding ratio for **QTP**:Al^3+^; each result is corroborated by two separate experiments from the following: NMR, absorbance or fluorescence studies. Overall, the sensor displays a marked selectivity for Al^3+^ over other ions explored in this study, including Cu^2+^. Results from this work provide rich insight into the binding mode and selectivity of the sensor. These findings will establish that the triazolyl–quinolinyl scaffold forms a well-defined, metal-selective binding pocket capable of differentiating Cu^2+^ from Al^3+^, positioning this platform as a strong candidate for next-generation metal-ion sensors, chelators, and coordination-driven functional materials. Our group is currently focusing on substituted pyridine and quinoline ion sensors. The scope of this manuscript is restricted to the design and evaluation of QTP with Cu^2+^ and Al^3+^ in comparison to a narrow selection of non-response-inducing cations. Consequently, to further develop this quinoline scaffold for practical applications, the impact on the photophysical response with a wider selection of cations, variation in the counter anions, and different conditions that mimic environmental pH will be investigated in future iterations of this work [43].

## Data Availability

Data related to investigations in this article are in the supplemental material. Additional raw data can be made available upon reasonable request.

## Supplementary Materials

The following supporting information can be downloaded at: https://www.mdpi.com/article/10.3390/s26144508/s1. The supplementary material consists of: Section A. General Experimental; Section B. Synthesis of 8-(4-phenyl-1H-1,2,3-triazol-yl)Quinoline (QTP); Section C. Spectra and Images; and Figure S1. (**A**) ^1^H-NNMR Spectrum of **QTP**. (**B**) 2D ^1^H-^1^H COSY Spectrum for **QTP**. (**C**) ^13^C-NMR Spectrum of **QTP**. Solvent: deuterated acetonitrile (CD_3_CN); Figure S2. Response of **QTP** (2.94 × 10^−4^ mol dm^−3^) treated with metal perchlorate salts (~2 × 10^−3^ mol dm^−3^) in acetonitrile under (**A**) UV-light (365 nm), (**B**) ambient light, and (**C**) paper strip practical applications: (i)—ambient light; (ii)—UV-light (365 nm)—pure QTP sensor; (iii)—UV-light (365 nm)—pure QTP sensor; QTP + Cu^2+^ and, QTP + Al^3+^. (**D**) Fluorescence spectra for **QTP** only (black) (2.94 × 10^−4^ mol dm^−3^), **QTP** with Fe^3+^ (red), Fe^2+^ (purple) and Al^3+^ (green) (concentration of perchlorate salts: ~3 × 10^−3^ mol dm^−3^); Figure S3. Normalized emission and fluorescence excitation spectra of QTP (2.94 × 10^−4^ mol dm^−3^) in acetonitrile (for the emission spectrum the excitation wavelength is 294 nm and for the excitation spectrum the monitored emission wavelength is 411 nm); Figure S4. Job’s plot of QTP with copper (II) perchlorate hexahydrate in acetonitrile based on absorbance monitored at 315 nm. $X=\frac{\left[QTP\right]}{\left[QTP+Cu2+\right]}$ for [QTP +Cu^2+^]: sum of molar concentrations of QTP and Cu^2+^, and [QTP]: molar concentration of QTP; Figure S5. Absorbance Titration with QTP and the perchlorate salt of Al^3+^; Figure S6. Stern–Volmer plot to determine the limit of detection (LOD) using the fluorescence output for **QTP** with Al^3+^ ion (observed wavelength: 415 nm). LOD = 4.27 µM; Figure S7. Job’s plot based on the ^1^H-NMR titration experiment with QTP and the perchlorate salt of Al^3+,^ observed chemical shift is for H-3; Figure S8. DFT Prediction for (**A**) Uncoordinated 2:1 **QTP**-Al^3+^ Complex and (**B**) Coordinated 2:1 **QTP**-Al^3+^ Complex; Figure S9. Detailed View of Optimized DFT Prediction for 2:1 **QTP**-Al^3+^ Complex; Figure S10. Benesi–Hildebrand plot for **QTP** with Cu^2+^ based on absorbance; Figure S11. Benesi–Hildebrand plot for **QTP** with Al^3+^ based on fluorescence.
