# Supplementary material for: Recognition of Cu2+ and Al3+ by a Quinolinyl 1,2,3-Triazole Chemosensor: A Comparative Study
Source: Sensors (Basel). 2026 Jul 15;26(14):4508. doi: 10.3390/s26144508 (PMC13416729; doi:10.3390/s26144508)
Supplement: Supplementary file 1 [file sensors-26-04508-s001.zip › sensors-4387673-supplementary.pdf]

Recognition of Cu^{2+} and Al^{3+} by a Quinolinyl 1,2,3-Triazole Chemosensor: A Comparative Study

Richard D. Govan [1], Tyler C. Camp [1], Vincent F. Hernandez [1], Precious Obiako [1], Debosreeta Bose[2], Debanjana Ghosh [3,*], Shainaz Landge [1,*] and Karelle Aiken [1,*]

[1] Department of Biochemistry, Chemistry, and Physics, Georgia Southern University, 521, College of Education Drive, Statesboro, GA 30460, USA

[2] Department of Basic Science and Humanities, Institute of Engineering and Management, University of Engineering and Management, New Town, Kolkata, West Bengal 700160, India; debosreeta.bose@iem.edu.in

[3] Department of Chemistry, Science Building West, Box—1652, Southern Illinois University Edwardsville, Edwardsville, IL 62026, USA

* Correspondence: slandge@georgiasouthern.edu (S.L.); kaiken@georgiasouthern.edu (K.A.); dghosh@siue.edu (D.G.)

Content	Page
General Experimental	2
Synthesis of 8-(4-phenyl-1H-1,2,3-triazol-1-yl)quinoline (QTP)	3
Figure S1– (A) ^{1}H -NMR, (B) 2D ^{1}H-^{1}H COSY & (C) ^{13}C-NMR spectra for QTP	4
Figure S2 – QTP with metal perchlorates under UV light (A) and ambient (B)	7
Figure S2 Photograph of paper strip practical applications (C)	7
Figure S2 Fluorescence spectra for QTP with Fe^{3+}, Fe^{2+} and Al^{3+} (D)	7
Figure S3 - Normalized emission and fluorescence excitation spectra of QTP	8
Figure S4 – Absorbance Job's Plot for QTP with Cu^{2+}	9
Figure S5 – Absorbance Titration for QTP with Al^{3+}	10
Figure S6 – Limit of Detection for Al^{3+}	11
Figure S7 – NMR Job's Plot for QTP with Al^{3+}	12
Figure S8 – Structures of sensor and sensor – Al^{3+} complex	13
Figure S9 – Optimized structure of sensor and Al^{3+}	13
Figure S10 – Benesi–Hildebrand plot for QTP with Cu^{2+} based on absorbance	14
Figure S11 – Benesi–Hildebrand plot for QTP with Al^{3+} based on fluorescence	15

A. General Experimental:

All chemicals and reactants were obtained through commercial sources (Alfa Aesar, OXCHEM, Sigma Aldrich, Acros, and Fisher) without further purification. Column chromatography was performed with Selecto Scientific Silica Gel (particle size 100-200 microns). NMR spectra were recorded on an Agilent MR4000DD2 spectrometer: 1H-NMR: 400 MHz and ^{13}C-NMR:100 MHz. Deuterated acetonitrile (CD_3CN). Signals were recorded in parts per million (*ppm*). References in the corresponding solvents were set according to residual CH_3CN at 1.94 ppm for ^{1}H-NMR and 1.32 ppm [$\underline{C}H_3$] and 118.26 [$\underline{C}N$] for ^{13}C-NMR. Signals for the ^{1}H-NMR multiplicity are described as: singlet (s), doublet (d), doublet of doublet (dd), triplet (t), multiplet (m), and coupling constants (*J,* Hz).

Absorption and steady-state fluorescence measurements were performed using a Shimadzu UV-2450 spectrophotometer and PerkinElmer LS55 with a well plate reader fluorimeter respectively. All spectroscopy experiments were performed at ambient temperature.

8-(4-Phenyl-1H-1,2,3-triazol-1-yl)quinoline (QTP),[1] a known molecule, was synthesized according to a previously reported procedure. A slight modification was made in the isolation of the crude product (*see below*).

B. Synthesis of 8-(4-phenyl-1H-1,2,3-triazol-1-yl)quinoline (QTP)[1]

Acetophenone tosylhydrazone (2.01 g, 6.94 mmol) and 8-aminoquinoline (2.00 g, 13.71 mmol) were suspended in toluene (70 ml). Pivalic acid (1.41 g, 13.71 mmol) was added to the reaction vessel, followed by the addition of copper (II) acetate anhydrous (1.26 g, 6.94 mmol). After 32 hours of stirring while refluxing the reaction was cooled and the solvent was removed in vacuo. The crude product was dissolved in acetonitrile (70 ml), then a concentrated aqueous solution of disodium ethylenediaminetetraacetate (EDTA) dihydrate (70 ml) was added to the organic suspension and stirred for 10 mins to remove the copper salt. The organic layer was removed, and the solvent was evaporated. Ethyl acetate (200 ml) was added to the crude product followed by washes with water (2 × 200 ml) and brine (2 × 200 ml), in that order. The ethyl acetate extract was dried over anhydrous sodium sulfate. The solvent was removed under vacuum and the resulting red-brown oil was purified using flash column chromatography (20% ethyl acetate in hexanes followed by 60% ethyl acetate in hexanes). Pure 8-(4-Phenyl-1H-1,2,3-triazol-1-yl)quinoline **(QTP)** was obtained as an off-white, beige powder 2.50 g (67%). The 1D ^{1}H- and ^{13}C-NMR spectra were consistent with those reported in the literature. **^{1}H NMR** (400 MHz, Acetonitrile-d_3) δ 9.04 (s, 1H), 9.00 (dd, J = 4.2, 1.7 Hz, 1H), 8.47 (dd, J = 8.4, 1.8 Hz, 1H), 8.25 (dd, J = 7.5, 1.4 Hz, 1H), 8.14 (dd, J = 8.3, 1.4 Hz, 1H), 8.00 (dd, J = 8.4, 1.3 Hz, 1H), 7.80 (dd, J = 8.3, 7.5 Hz, 1H), 7.65 (dd, J = 8.4, 4.2 Hz, 1H), 7.52 (t, J = 7.6 Hz, 1H), 7.45 – 7.37 (tt, J = 7.4, 1.3 Hz, 1H). **^{13}C NMR** (101 MHz, Acetonitrile-d_3) δ 151.5, 146.7, 140.7, 136.7, 133.9, 131.0, 129.7, 129.2, 129.1, 128.2, 126.4, 125.6, 125.1, 124.4, 122.6.

C. Spectra and Images

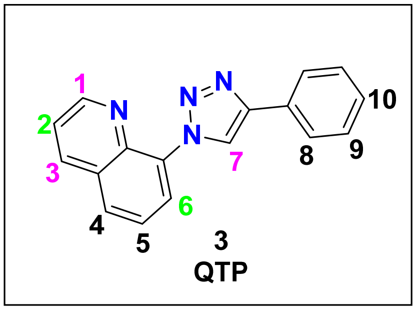

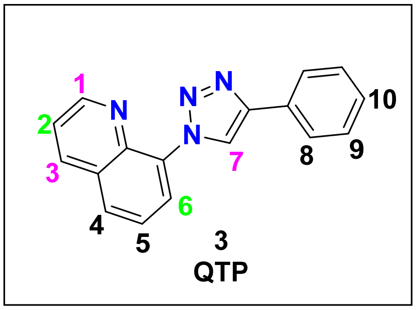

(A)

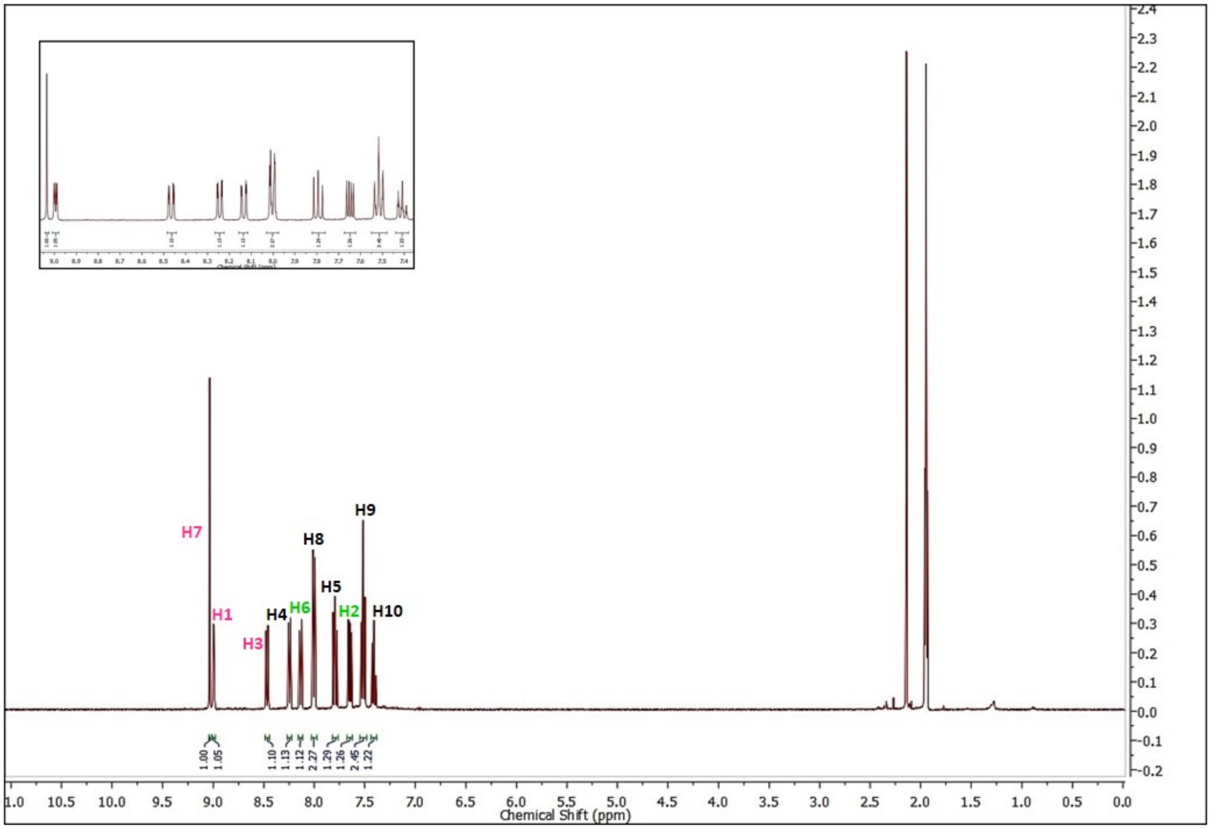

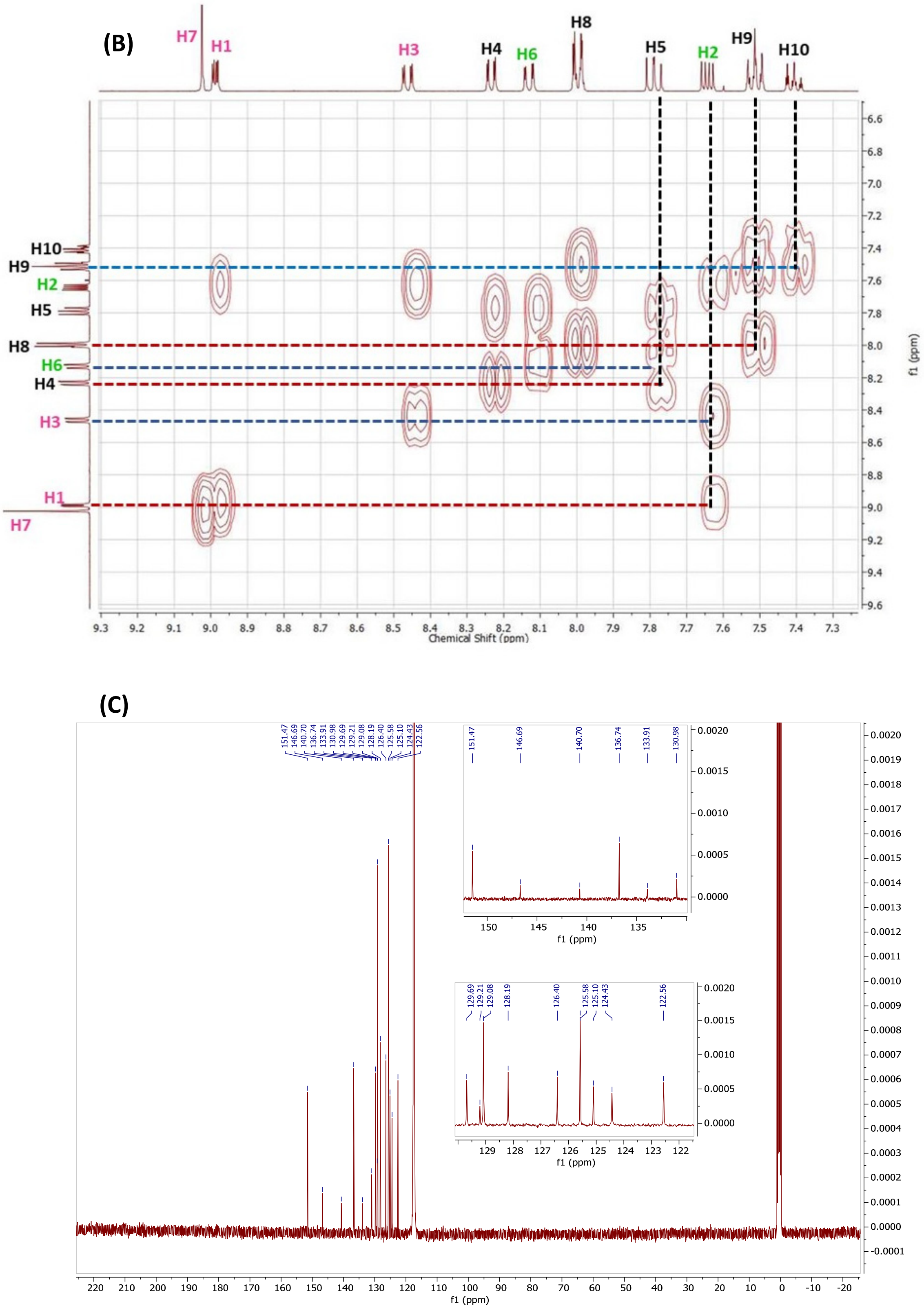

Figure S1. (A) ^{1}H-NMR Spectrum of **QTP**. **(B)** 2D ^{1}H-^{1}H COSY Spectrum for **QTP**. **(C)** ^{13}C-NMR Spectrum of **QTP**. Solvent: deuterated acetonitrile (CD_3CN).

The 2D ^{1}H-^{1}H COSY experiment in CD_3CN allowed us to unambiguously assign resonances to each hydrogen in the molecule (Figure S1). The triazole Csp^2-H (H-7), not coupled to any other proton, produces a distinct singlet at 9.04 ppm. A doublet (*d*) at 8.99 ppm is due to the quinoline's H-1 and shows a clear correlation to the 7.65 ppm doublet of doublets (*dd*) for H-2. The latter proton, H-2, also neighbours H-3 at 8.64 ppm (*d*). The rest of the quinoline hydrogens, H-4, H-5 and H-6, occur at 8.24 ppm (*d*), 7.65 ppm (*dd*) and 8.14 ppm (*d*), respectively. The two correlations for H-5 with H-4 and H-6 are quite distinct in the 2D spectrum. The phenyl's resonances are found at 8.01 ppm (*d*) for H-8, 7.52 ppm (*dd* or apparent triplet (*t*)) for H-9 and 7.41 ppm (*t*) for H-10.

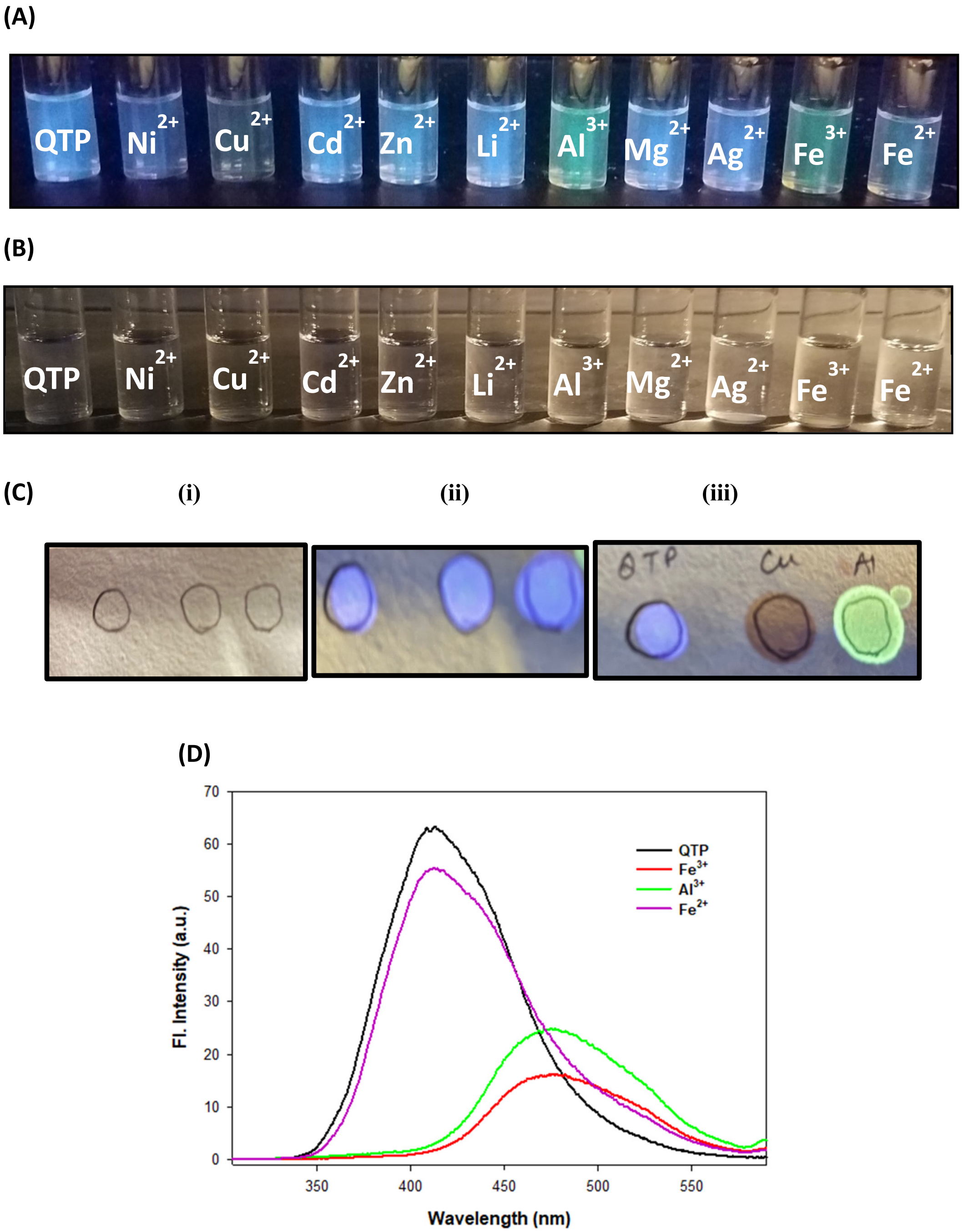

Figure S2. Response of **QTP** (2.94×10^{-4} mol dm^{-3}) treated with metal perchlorate salts ($\sim 2 \times 10^{-3}$ mol dm^{-3}) in acetonitrile under **(A)** UV-light (365 nm), **(B)** ambient light, and **(C)** paper strip practical applications: (i) ambient light; (ii) UV-light (365 nm) pure QTP sensor; (iii) UV-light (365 nm) pure QTP sensor; QTP + Cu^{2+} and, QTP + Al^{3+}. **(D)** Fluorescence spectra for **QTP** only (black) (2.94×10^{-4} mol dm^{-3}), **QTP** with Fe^{3+} (red), Fe^{2+} (purple) and Al^{3+} (green) (concentration of perchlorate salts: $\sim 3 \times 10^{-3}$ mol dm^{-3}). *Exc*. at 294 nm.

Fe^{2+} and Al^{3+} induce similar responses with **QTP**. The fluorescence spectra (Fig. S2C) also show similar λ_{max} for both cations with output intensity that is ~25% and ~40% of that of **QTP** for Fe^{2+} and Al^{3+}, respectively.

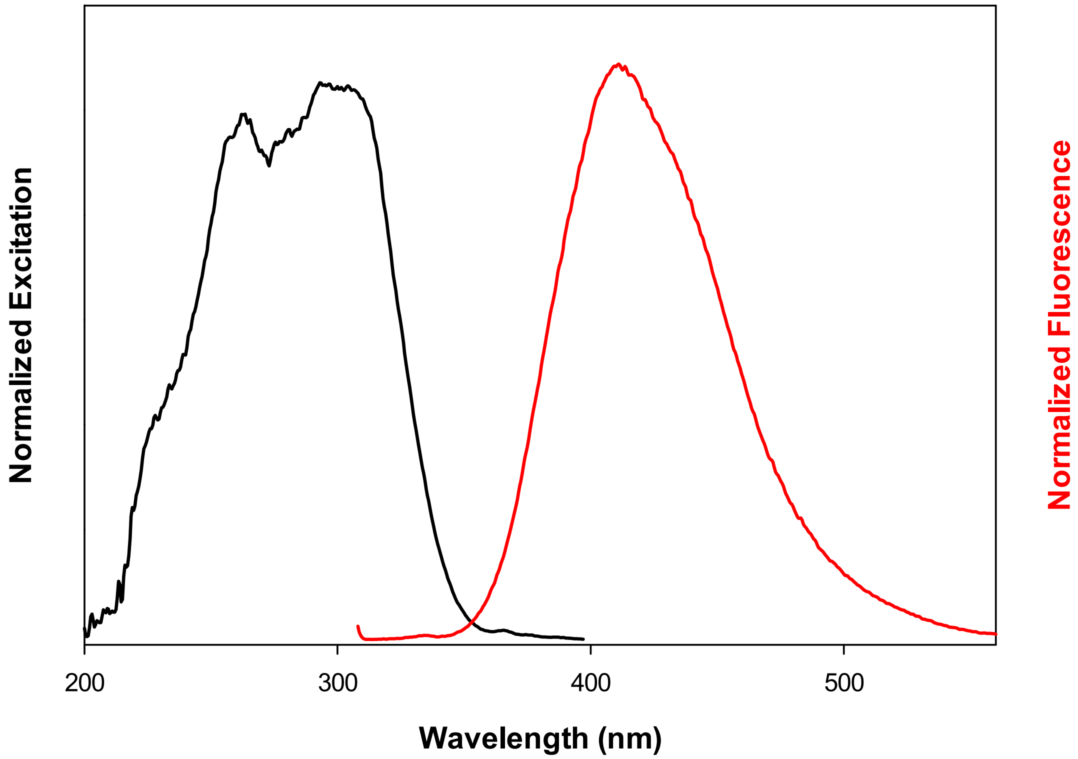

Figure S3. Normalized emission and fluorescence excitation spectra of QTP (2.94×10^{-4} mol dm^{-3}) in acetonitrile (for the emission spectrum the excitation wavelength is 294 nm and for the excitation spectrum the monitored emission wavelength is 411 nm).

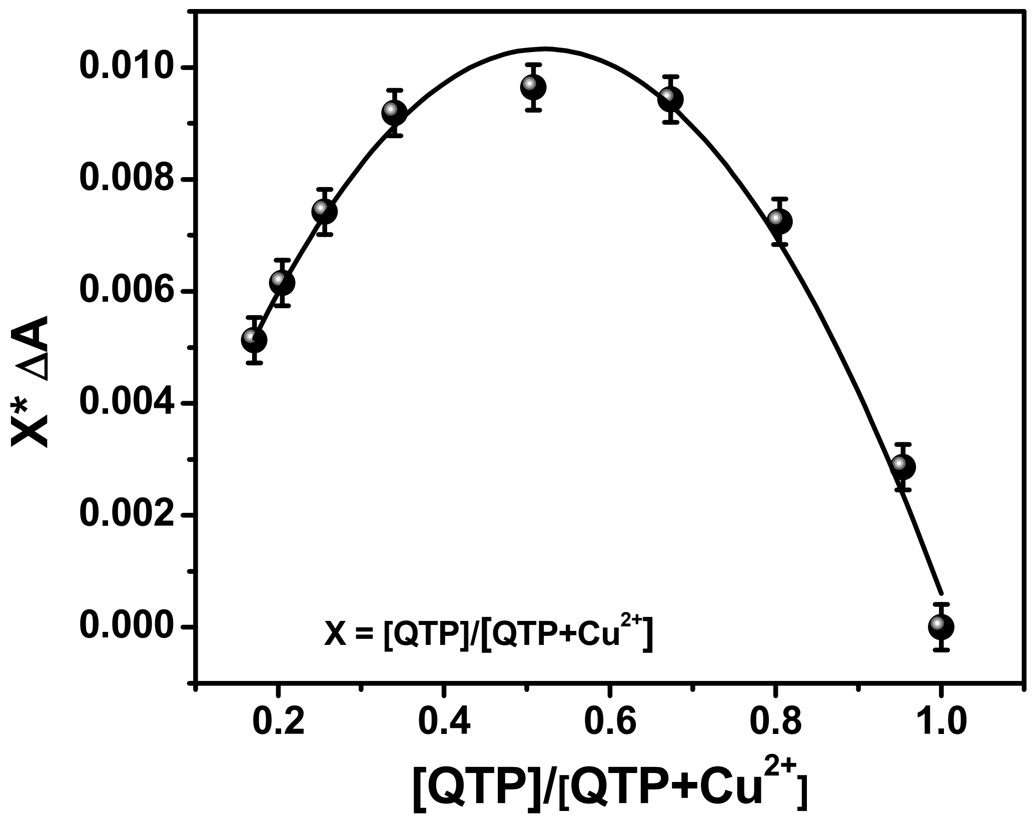

Figure S4. Job's plot of **QTP** with copper (II) perchlorate hexahydrate in acetonitrile based on absorbance monitored at 315 nm. $X = \frac{[\mathbf{QTP}]}{[QTP+Cu2+]}$ for [QTP $+Cu^{2+}$]: sum of molar concentrations of **QTP** and Cu^{2+}, and [**QTP**]: molar concentration of **QTP**.

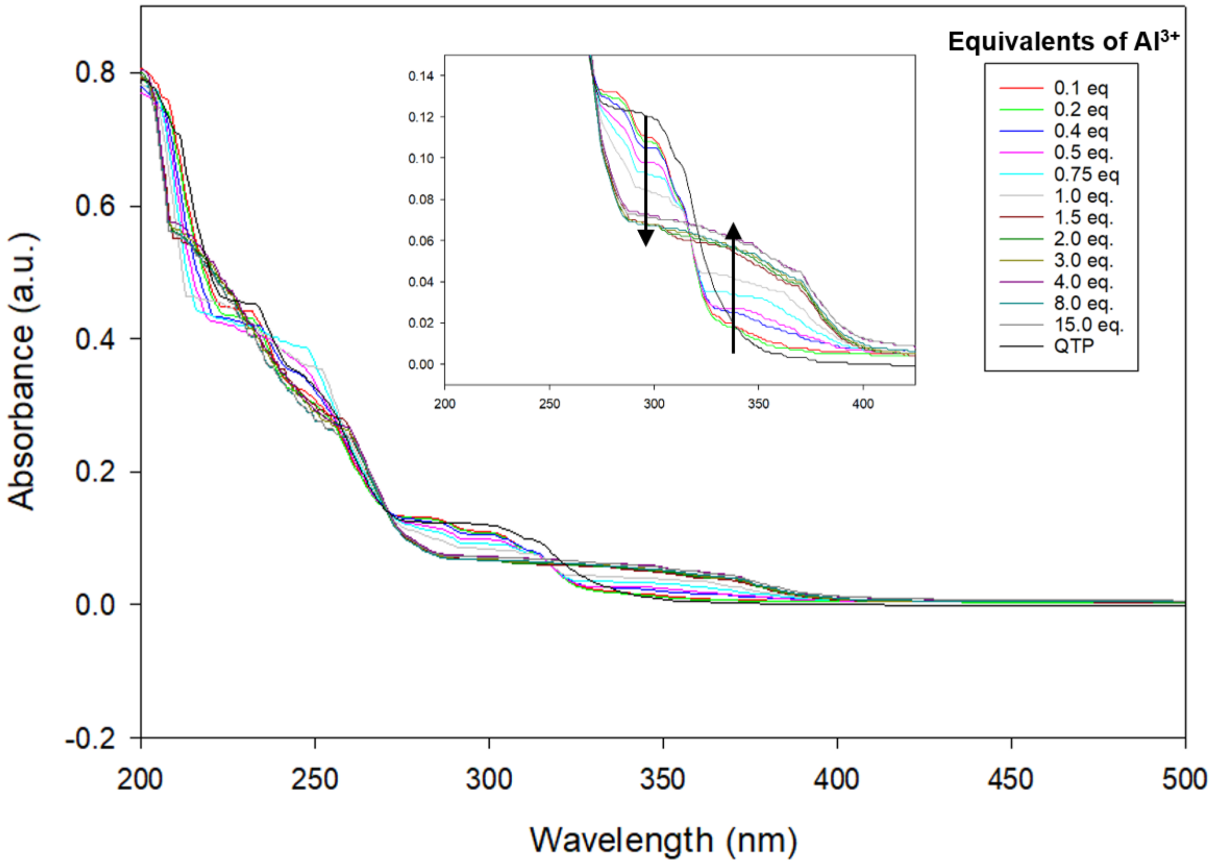

Figure S5. Absorbance titration with QTP and the perchlorate salt of Al^{3+}.

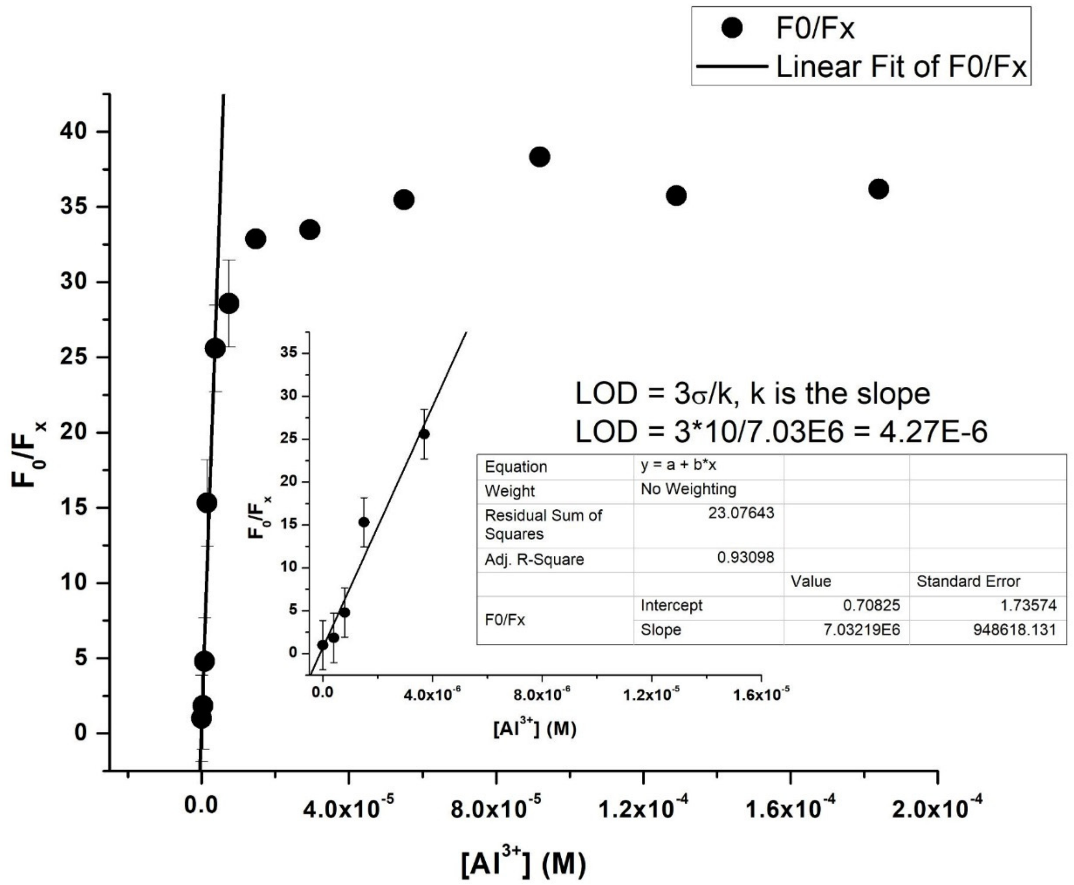

Figure S6. Stern–Volmer plot to determine the limit of detection (LOD) using the fluorescence output for **QTP** with Al^{3+} ion (observed wavelength: 415 nm). LOD = 4.27 µM.

The detection limit of **QTP** for Al^{3+} was calculated using the fluorescence titration data according to the IUPAC definition. To find the slope, the ratio of fluorescence intensities (F_0 / F_x) at 415 nm was plotted against [Al^{3+}]. A limit of detection (LOD) of 4.27×10^{-6} mol dm^{-3} was obtained with a linear range of 4×10^{-7} to 7.4×10^{-6} mol dm^{-3} of Al^{3+} concentration.

$$\text{Limit of Detection (LOD)} = 3\sigma/k \qquad \text{Equation (1)}$$

where k is the slope of the plot and sigma is the number of iterations.

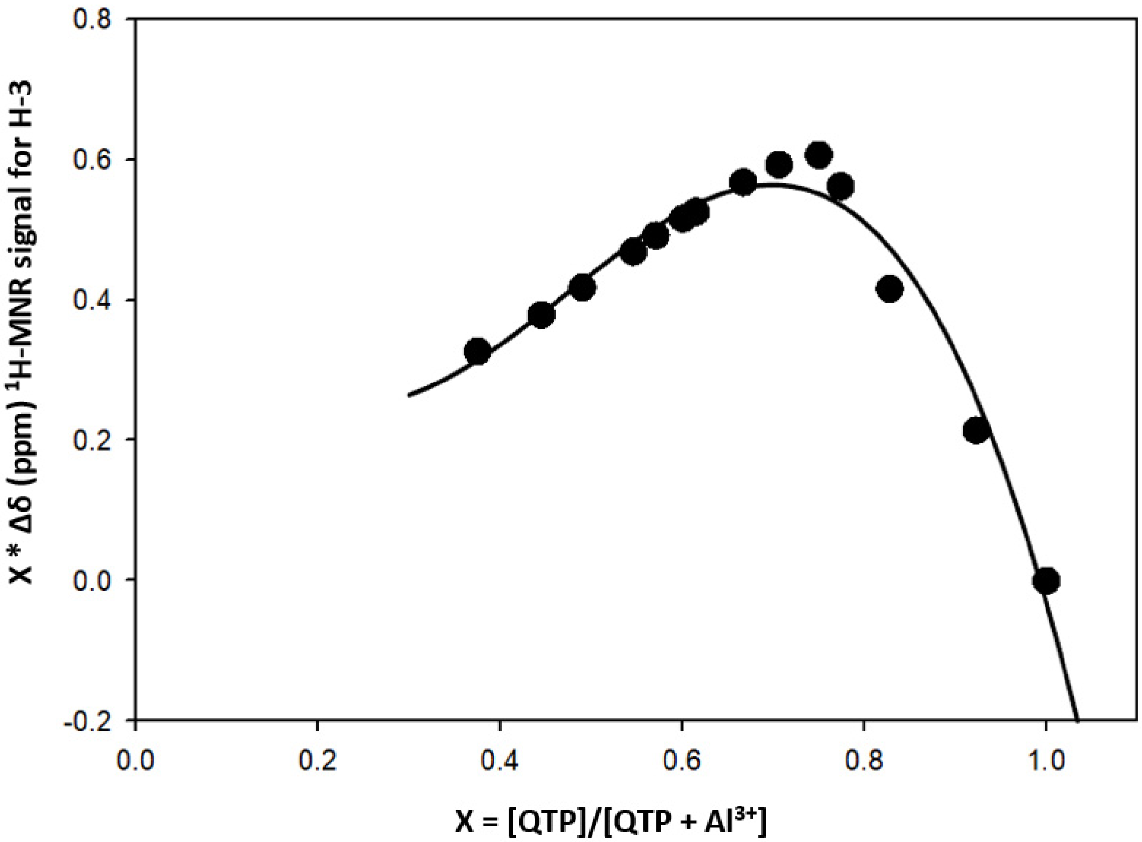

Figure S7. Job's plot based on a ^{1}H-NMR titration experiment with QTP and the perchlorate salt of Al^{3+}: the observed chemical shift is for H-3.

(A) (B)

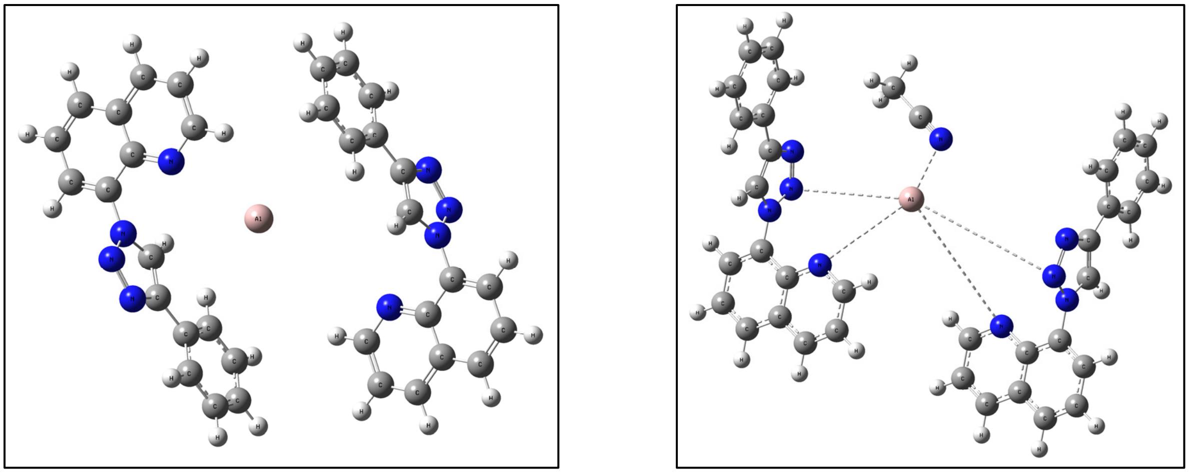

Figure S8. DFT prediction for **(A)** uncoordinated 2:1 **QTP**-Al^{3+} complex and **(B)** coordinated 2:1 **QTP**-Al^{3+} complex.[2]

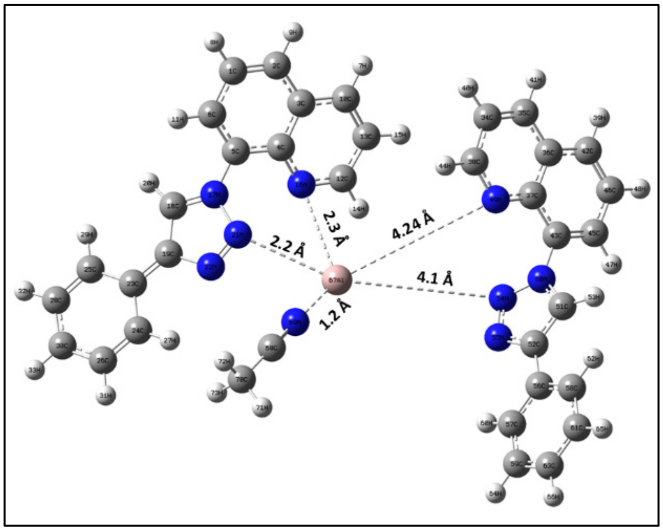

Figure S9. Detailed view of optimized DFT prediction for 2:1 **QTP**-Al^{3+} complex.[2]

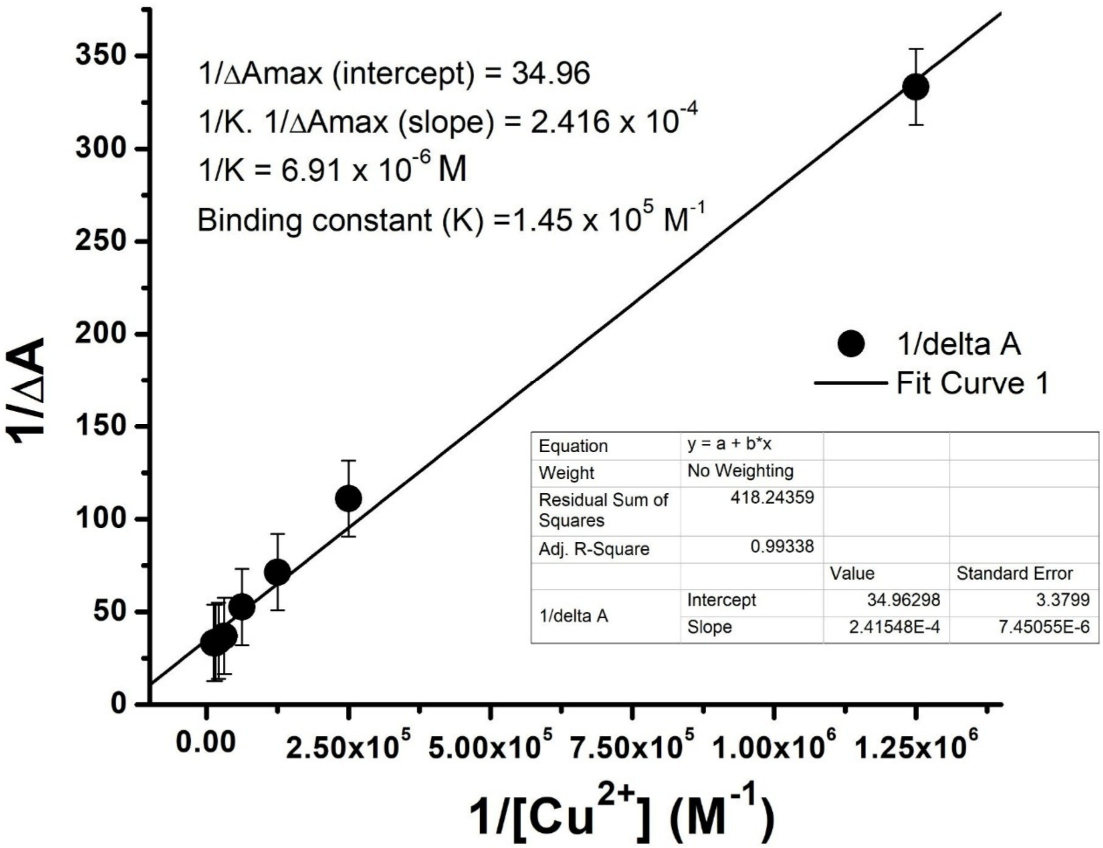

Figure S10. Benesi–Hildebrand plot for **QTP** with Cu^{2+} based on absorbance.

1/ΔA was graphed against $1/[Cu^{2+}]$ using Equation 1, which yielded a linear plot, and from the slope the binding constant (K) was determined.[3]

$$1/\Delta A = 1/\Delta A_{max} + 1/K.\ 1/\ \Delta A_{max}.\ 1/[Cu^{2+}] \qquad \text{Equation (2)}$$

where $\Delta A = A_x - A_0$; $\Delta A_{max} = A_\infty - A_0$. A_0, A_x and A_∞ are the absorbances of **QTP** at 316 nm in the absence, at an intermediate concentration and at a concentration of complete interaction of **QTP** with Cu^{2+}, respectively.

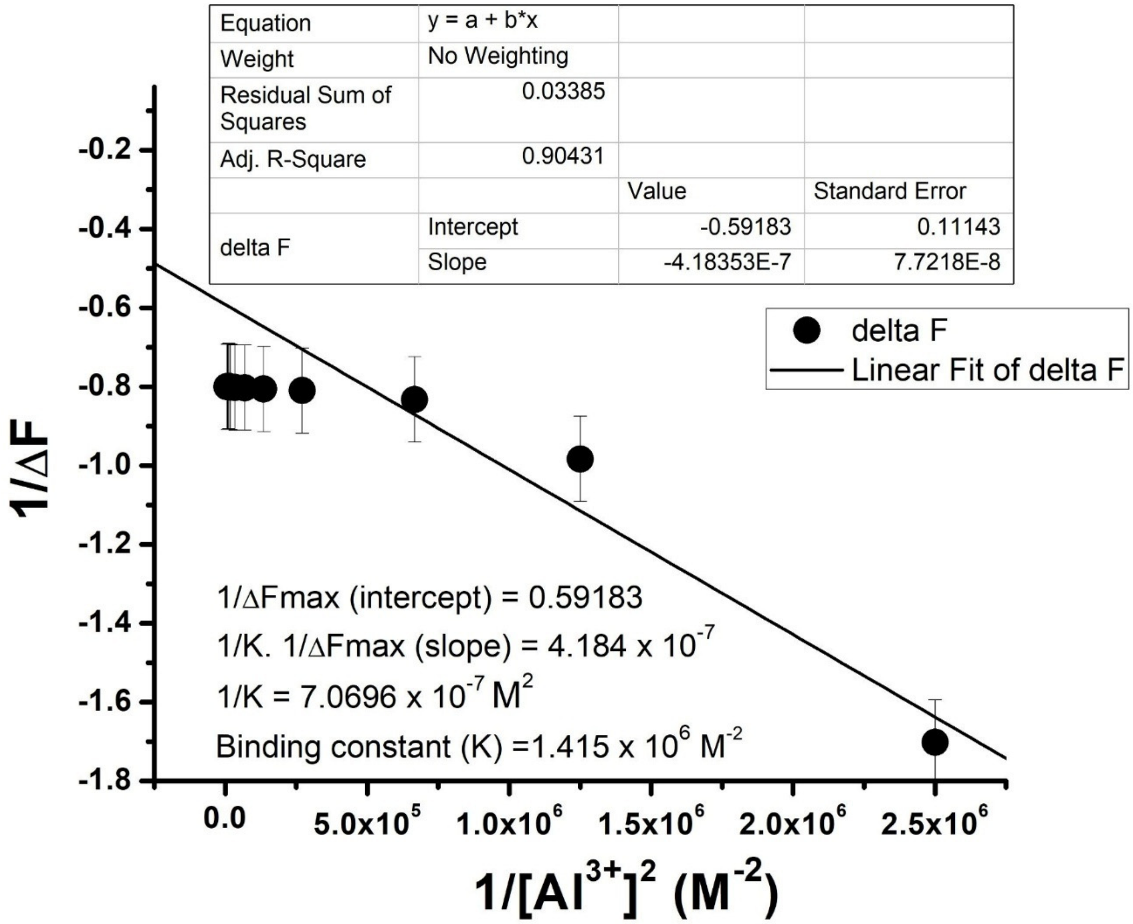

Figure S11. Benesi–Hildebrand plot for **QTP** with Al^{3+} based on fluorescence.

1/ΔF was graphed against $1/[Al^{3+}]^2$ using Equation 1, which yielded a linear plot, and from the slope the binding constant (K) was determined.[3]

$$1/\Delta F = 1/\Delta F_{max} + 1/K.\ 1/\ \Delta F_{max}.\ 1/[Al^{3+}]^2 \qquad \text{Equation (3)}$$

where $\Delta F = F_x - F_0$; $\Delta F_{max} = F_\infty - F_0$. F_0, F_x and F_∞ are the emission of **QTP** at 410 nm in the absence, at an intermediate concentration and at a concentration of complete interaction of **QTP** with Al^{3+}, respectively.

(1) Chen, Z.; Yan, Q.; Liu, Z.; Xu, Y.; Zhang, Y. Copper-Mediated Synthesis of 1,2,3-Triazoles from N-Tosylhydrazones and Anilines. *Angewandte Chemie* **2013**, *125* (50), 13566-13570. DOI: 10.1002/ange.201306416 (accessed 2018/06/26).
(2) Frisch MJ, Trucks GW, Schlegel HB, Scuseria GE, Robb MA, Cheeseman JR, Scalmani G, Barone V, Mennucci B, Petersson GA, Nakatsuji H, Caricato M, Li X, Hratchian HP, Izmaylov AF, Bloino J, Zheng G, Sonnenberg JL, Hada M, Ehara M, Toyota K, Fukuda R, Hasegawa J, Ishida M, Nakajima T, Honda Y, Kitao O, Nakai H, Vreven T, Jr. JAM, Peralta JE, Ogliaro F, Bearpark M, Heyd JJ, Brothers E, Kudin KN, Staroverov VN, Kobayashi R, Normand J, Raghavachari K, Rendell A, Burant JC, Iyengar SS, Tomasi J, Cossi M, Rega N, Millam JM, Klene M, Knox JE, Cross JB, Bakken V, Adamo C, Jaramillo J, Gomperts R, Stratmann RE, Yazyev O, Austin AJ, Cammi R, Pomelli C, Ochterski JW, Martin RL, Morokuma K, Zakrzewski VG, Voth GA, Salvador P, Dannenberg JJ, Dapprich S, Daniels AD, Farkas Ö, Foresman JB, Ortiz JV, Cioslowski J, Fox DJ (2009). Gaussian Inc., Wallingford CT.
(3) Nunes, M. C.; dos Santos Carlos, F.; Fuganti, O.; Galindo, D. D. M.; De Boni, L.; Abate, G.; Nunes, F. S. Turn-on fluorescence study of a highly selective acridine-based chemosensor for Zn2+ in aqueous solutions. *Inorganica Chimica Acta* **2020**, *499*, 119191. DOI: https://doi.org/10.1016/j.ica.2019.119191.
